# Numerical Simulation Analysis of the Hydrogen-Blended Natural Gas Leakage and Ventilation Processes in a Domestic House

**DOI:** 10.1021/acsomega.3c03551

**Published:** 2023-09-13

**Authors:** Minghao Li, Shuangqing Chen, Weidong Jiang, Yuchun Li, Zhe Xu, Bing Guan, Xingwang Wang, Xiaoqiang Lin, Tianqing Liu

**Affiliations:** † School of Petroleum Engineering, Northeast Petroleum University, Daqing, Heilongjiang Province 163318, China; ‡ Post-doctoral Programme, Daqing Oilfield Co. Ltd., Daqing, Heilongjiang Province 163453, China; ¶ Anda Qingxin Oil Field Development Co. Ltd., Anda, Heilongjiang Province 151413, China; § Daqing Oilfield Design Institute Co. Ltd., Daqing, Heilongjiang Province 163453, China; ∥ Gas Technology Institute of Petrochina Kunlun Gas Co. Ltd., Harbin, Heilongjiang Province 150001, China; ⊥ Institute of Unconventional Oil and Gas, Northeast Petroleum University, Daqing, Heilongjiang Province 163318, China; # Exploration and Production Research Institute, SINOPEC, Beijing 100083, China

## Abstract

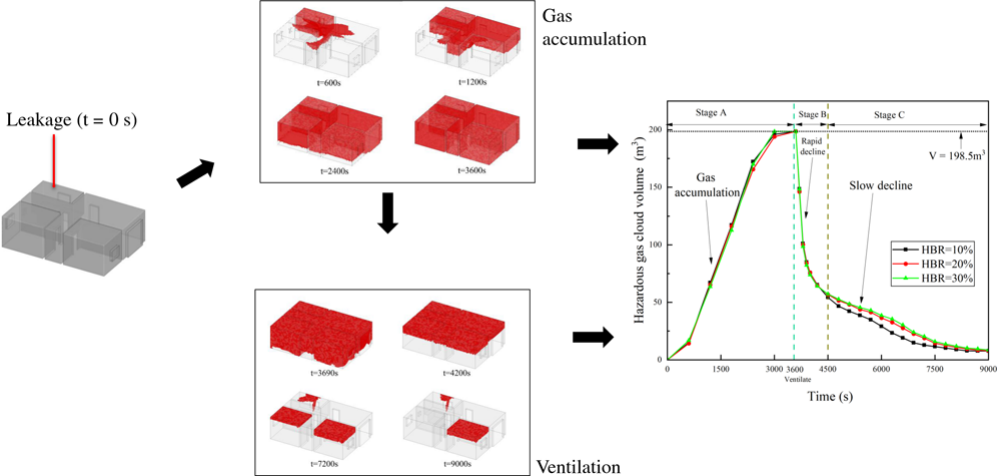

The blending of hydrogen in natural gas may have effects on the safety of its usage in a domestic house. In this work, the leakage accident of hydrogen-blended natural gas (HBNG) in the kitchen of a domestic house is analyzed by CFD with a hydrogen blending ratio (HBR) ≤ 30%. The whole process is divided into the gas accumulation process and the ventilation process. In the initial leakage stage, the influence of heights and the HBR on the gas distribution is analyzed. HBNG concentration increases with increasing height. Based on the exit Froude number, the formation of a gas cloud in the kitchen is significantly influenced by the initial momentum and buoyancy, while it is more driven by the concentration gradient beyond the kitchen. In contrast to height, the variation of HBR on the HBNG distribution is not significant. In the ventilation process, the evolution of the hazardous gas cloud volume is analyzed. With windows and doors closed, the hazardous gas cloud fills the house in approximately 3600 s after the leakage occurs. When windows and doors are open for ventilation, the volume of the hazardous gas cloud first declines rapidly and then slowly. The reasons for the variation rate of hazardous gas cloud volume are analyzed according to ventilation conditions. The difference during the decline stage for different HBRs is analyzed according to the gas layering properties. Under a lack of convection condition, the ventilation process finally reaches a stagnant stage. In addition, another ventilation process has been investigated after extending the gas accumulation time. After extending the gas accumulation time, the effect of different HBRs on the ventilation process remains the same as before. However, it postpones the time point to enter the stagnation stage. As gas accumulation time extends from 3600 to 5400 and 7200 s, the ventilation time into the stagnation stage increases from about 4800 to 5400 and 6000 s, respectively. This study has implications for the establishment of a risk assessment system based on hazardous gas cloud volume.

## Introduction

Hydrogen (H_2_) is one of the zero carbon emission fuels that is recognized as a significant new energy source worldwide.^1^ However, the technology of storage and transportation of pure H_2_ has not been completely established. Pipeline transportation is a suitable way to balance the economy and efficiency. However, hydrogen embrittlement occurs in normal steel pipelines when storing and transporting H_2_ under high pressure.^2^ It may lead to pipeline failures.^3,4^ The one-time investment is large for laying special H_2_ pipelines, and the construction cost is 200% higher than a natural gas pipeline with the same transportation capacity.^5^ Therefore, using the existing natural gas pipeline for mixed natural gas and H_2_ transportation is a possible choice.^6^ Hydrogen-blended natural gas (HBNG) as a fuel can both significantly reduce carbon emissions and avoid large construction costs. With a reasonable proportion of H_2_ blending, it has been proven to be fully feasible without significant risk of pipeline failure. In countries such as the Netherlands, USA, France, UK, and China, some small-scale practical projects have been realized for domestic consumers, as shown in Table 1.^7^ With the feasibility of HBNG gradually proven, many works have been carried out on the reliability of HBNG at the domestic consumer level.

**Table 1:** HBNG Project in Different Countries

country	year	project/company name
France	2018	GRHYD
German	2019	Avacon
China	2019	SPIC
UK	2020	HyDeploy

Safety is an important issue in the HBNG application. H_2_ has a smaller minimum ignition energy, a larger explosive limit range, and a lower density^8^ than CH_4_, resulting in a high risk of explosion.^9^ Domestic buildings have poor ventilation and are prone to explosive accidents. Research on the HBNG leakage process is necessary to design effective prevention and emergency response strategies.

Due to the risk of H_2_, full-scale leakage experiments are difficult to conduct. A common replacement method is numerical simulation based on computational fluid dynamics (CFD). The reliability has been verified by experiments.^10^ Li et al. conducted CFD numerical simulations to research the diffusion behavior of hydrogen-rich natural gas at a hydrogen-natural gas mixing station.^11^ The effects of HBR, leakage rate, wind direction, wind speed, and other factors on the formation of a flammable gas cloud were analyzed. Sun et al. conducted CFD simulations for HBNG leakage in a semienclosed space to study the diffusion characteristics with different wind speed magnitudes and HBR.^12^ It was found that higher wind speed prevents upward diffusion and accelerates its accumulation in the horizontal direction. Some typical scenarios of HBNG leakage processes have also been simulated by the CFD method to analyze the diffusion characteristics and the evolution of hazardous areas.^13−16^ In these studies, Jia et al. and Sun et al. analyzed the response time and arrangement plans for the leak alarm based on CFD simulation results.^13,15^ Li et al. found that the thickness of the flammable area formed by HBNG leakage under confined conditions may be higher than that of H_2_ leakage.

The above research has established some basis for the safety aspects of HBNG, but there are still some unclear and worth mentioning issues. The effect of differences between H_2_ and CH_4_ physical properties on flammable gas clouds needs to be further studied. The experiments in a closed space conducted by Marangon and Carcassi found that layering characteristics appeared when a CH_4_ and H_2_ mixture is released.^17^ In addition, the analysis of the H_2_ leakage process found that low-pressure leakage conditions may result in the faster formation of hazardous gas clouds.^18^ For HBNG leakage, it is unclear whether a similar phenomenon would occur if HBR is sufficiently high. The pipe network system, obstacles, and ventilation conditions in domestic houses are complex. In some dated houses, it is difficult to cut off the gas supply after a leakage accident. It leads to a higher risk of fire and explosion accidents. Few works have adequately analyzed the process of forming hazardous gas clouds in whole HBNG leakage and ventilation processes under these conditions.

In this work, CFD numerical simulations of HBNG leakage and ventilation in a typical home were conducted. The evolution processes of mixture gas distribution, hazardous gas cloud volume under gas accumulation, and the ventilation process were studied. The effects of height and HBR on the gas distribution were analyzed. The gas accumulation and ventilation processes after leakage were characterized according to the different stages. Based on the results of the analysis, some recommendations for reducing risk were proposed.

## Methods

**Figure 1:**
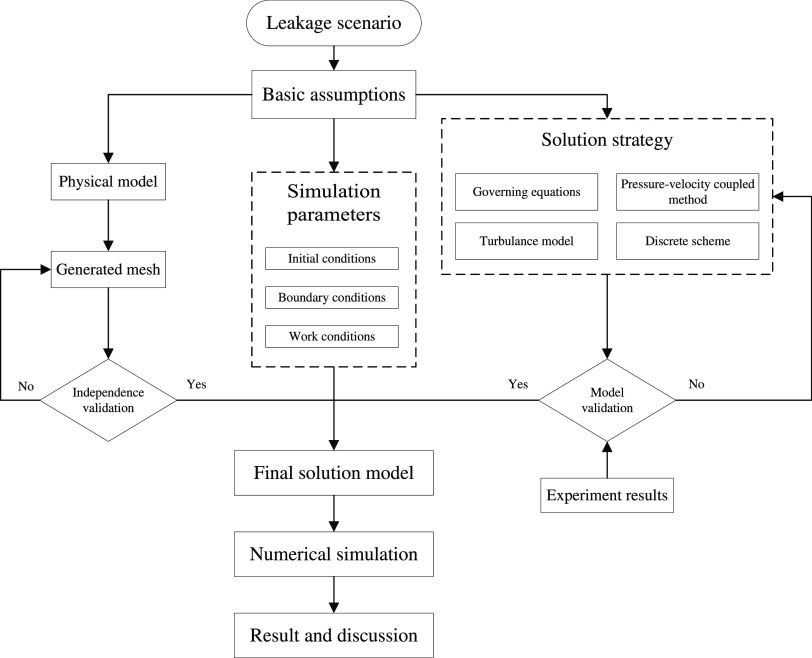
Flowchart of the building solution model.

In this work, it is important to build a reasonable solution model. It needs to be built from a real leakage scenario and based on reasonable assumptions and simplifications. The physical model, mathematical model, initial state, boundary conditions, and other simulation details need to be established. Numerical methods and mesh independence also need to be validated. To ensure that the solution model is sufficiently reliable, a workflow is established, as shown in Figure. 1.

### Physical Model

**Figure 2:**
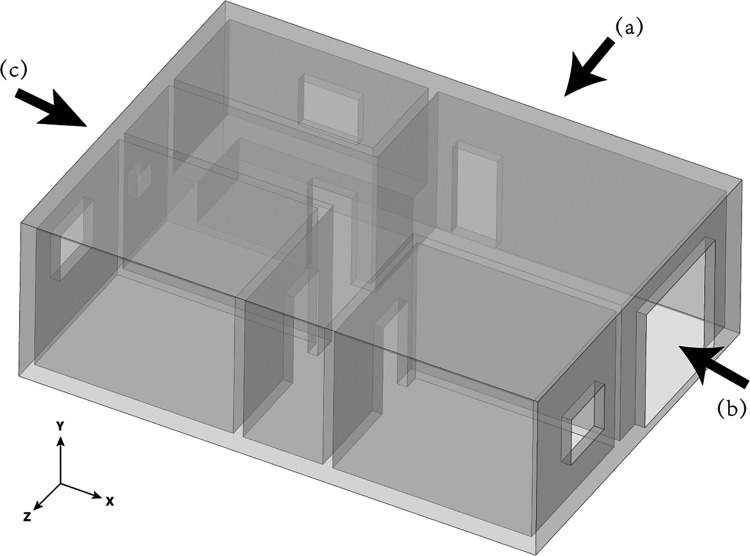
Schematic diagram of the domestic house.

**Figure 3:**
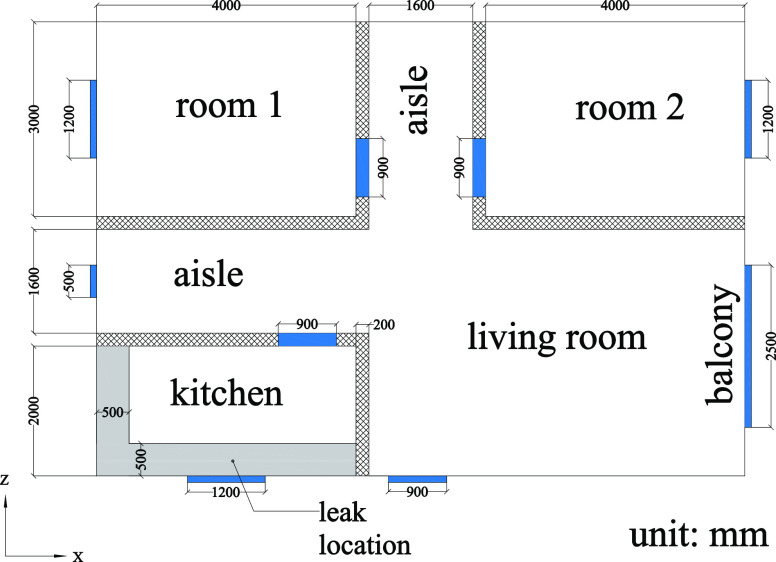
Layout of a domestic house for numerical simulations.

**Figure 4:**
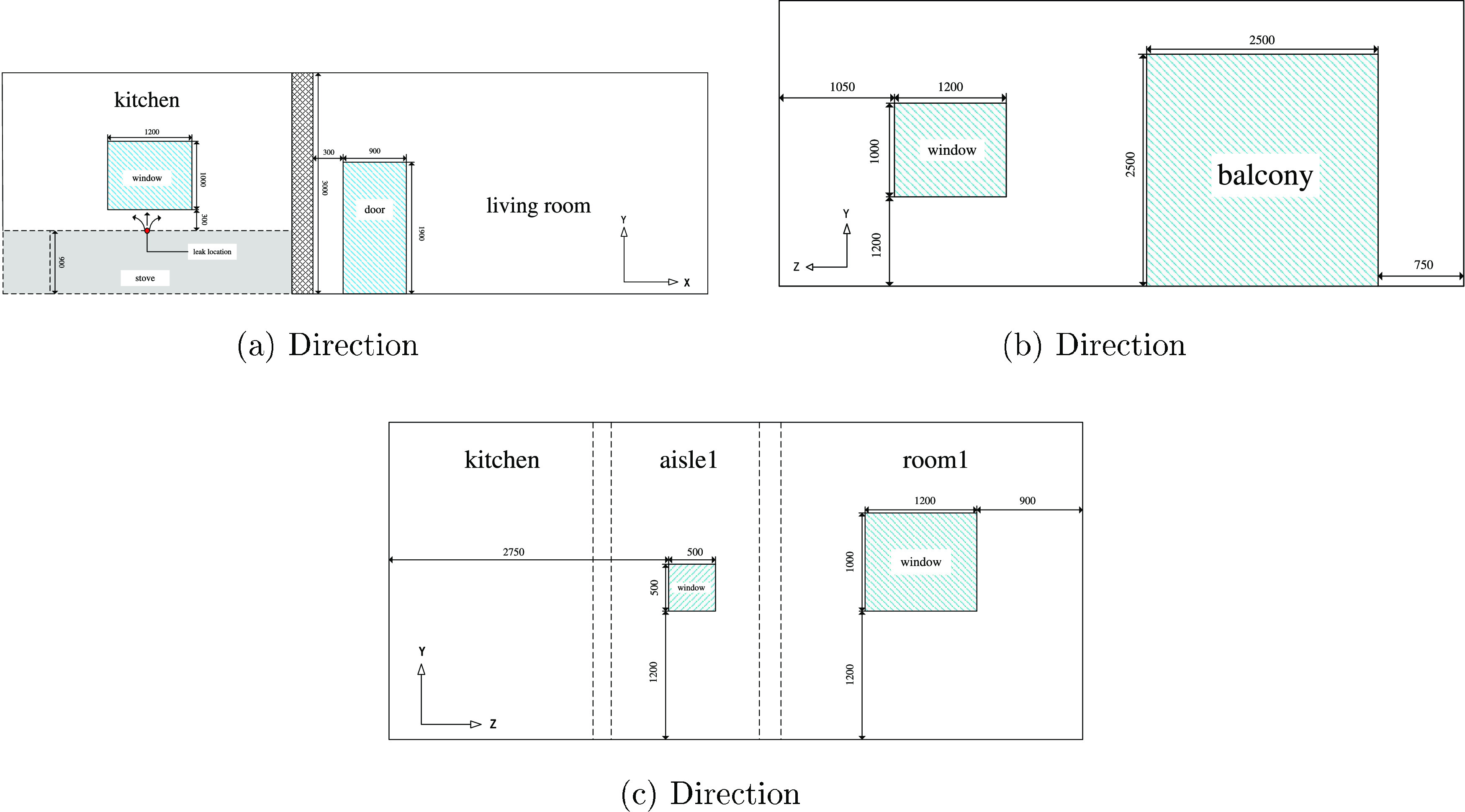
Geometry size of different directions in Figure 2.

The leakage scenario chosen in this work is a normal domestic house with a kitchen. It is shown in Figure 2. The various objects placed in the house are ignored to simplify the research. The basic layout of the domestic house is shown in Figure 3(unit: mm). The blue areas indicate doors and windows, which can be opened and closed as ventilation boundaries. The shadow and gray areas indicate the wall and the stove, respectively. In China, the inner diameter of the hose for a built-in gas stove is normally 9.5 mm. To simulate an HBNG leakage in this house, a 9.5 mm diameter leakage hole is considered on the stove in the gray area. The detailed geometry size of the doors, windows, and balcony in the (a), (b), and (c) directions are shown in Figure 4(unit: mm), where the height of the house is 3 m, and the interior volume of the whole house is 198.5 m^3^. The red circle in (a) indicates the leakage hole, which is 0.9 m above the floor.

### Mathematical Model

#### Assumptions and Simplifications

Several necessary assumptions and simplifications are made before establishing the CFD model for numerical simulations. (1) Leaked HBNG does not react with air in a chemical reaction at indoor temperature. (2) Pressure fluctuations in the gas pipeline system are ignored. (3) The flow rate of HBNG is constant in the whole process. (3) HBNG is considered a mixture of H_2_ and CH_4_ only. The HBR means the volume fraction. For example, HBR = 10% means that the HBNG consists of 10% volume of H_2_ and 90% of CH_4_. (4) The doors and windows of the house ignore gaps when they are closed. Consider the house as a completely enclosed space when the doors and windows are closed. (5) The gases in this work are considered to be compressible ideal gases.

The following analysis was made concerning the identification of (5). Some research studies consider flows (<0.3 Ma) as incompressible.^19,20^ The pressure in commonly used indoor gas pipes and appliances is approximately 2000–3000 Pa.^21^ According to the hole leakage model^22^ and numerical simulation test, the gas velocity when 100% CH_4_ leaks from a 9.5 mm diameter hole is about 80 m/s close to 0.25 Ma. It is close to the cutoff value for compressible and incompressible flow. In order to ensure accuracy as much as possible, it is reasonable to consider the flow in this work as compressible.

#### Governing Equations

Based on the above assumptions and simplifications, the governing equations for gas flow in this research contain the conservation of mass, momentum, and energy equations and the component equation.

The mass conservation (continuity) equation is shown in eq 1.
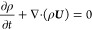
1

The momentum conservation equation is shown in eq 2.
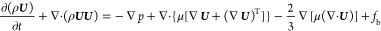
2

The meaning of the UU representation is as follows.
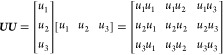


The component conservation equation is shown in eq 3.
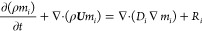
3

The energy conservation equation is shown in eq 4.
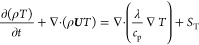
4

#### Turbulence Model

The standard *k* – ϵ model is a fairly stable and well-established turbulence model that is widely used in a variety of applications including gas diffusion.^23^ The *k* and ϵ equations are shown in eqs 5 and 6 in the standard *k* – ϵ model. In this work, a semiempirical method of wall functions is applied for the near-wall treatment in order to reduce computational costs. The scalable wall function is selected because of its greater flexibility than the standard wall function and less cost than the enhanced wall function. When generating prismatic layer meshes for flow-critical areas, one must ensure that the first layer is in the log-law region.
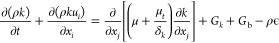
5
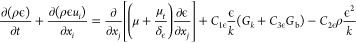
6

For the standard *k* – ϵ model, the core is the turbulent viscosity μ_*t*_ expressed in terms of turbulent kinetic energy *k* and the turbulent dissipation rate ϵ*·*μ_*t*_ calculated by eq 7.
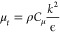
7
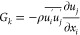
8

The effect of buoyancy on turbulence needs to be considered; it is defined as *G*_b_ calculated by eq 9. The effect of buoyancy on the turbulent dissipation rate is shown by *C*_3_ calculated by eq 10.
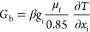
9
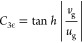
10

*C*_1ϵ_, *C*_2ϵ_, δ_*k*_, δ_ϵ_, and *C*_μ_ are constants in the standard *k* – ϵ model selected, as shown in Table 2. These values have been determined and validated by turbulent flow experiments including jet flow.

**Table 2:** Constants in the Standard *k* – *ϵ* Model

model constant	*C*_1ϵ_	*C*_2ϵ_	δ_*k*_	δ_ϵ_	*C*_μ_
value	1.44	1.92	1.0	1.3	0.09

### Simulation Details

#### Initial Conditions

For the initial state of the simulation (*t* = 0 s), the computational domain is filled with air. The initial concentration and momentum of HBNG are zero. The initial temperature in the house is assumed to be set as 298 K, and the initial gauge pressure is 0 Pa (absolute pressure = 101 325 Pa).

#### Boundary Conditions

In this work, gas accumulation and ventilation processes with no cutoff of the HBNG gas source are studied. The boundary conditions at the door, window, and balcony vary in different processes, as shown in Table 3. Based on previous assumptions, the flow is considered compressible; therefore, the leak hole is selected to be set as the mass flow inlet. The doors, windows, and balcony are set as no-slip walls in the gas accumulation process and as pressure outlets with gauge pressure = 0 Pa in the ventilation process.

**Table 3:** Definition of Boundary Condition Types

boundary names	gas accumulation process	ventilation process
leak hole	mass flow inlet	mass flow inlet
window	no-slip wall	pressure outlet
door	no-slip wall	pressure outlet
balcony	no-slip wall	pressure outlet
wall	no-slip wall	no-slip wall

#### Gas Physical Properties

The lower explosion limit (LEL) and upper explosion limit (UEL) are important in the safety analysis of flammable gases. The physical properties of CH_4_ and H_2_ are shown in Table 4.

**Table 4:** Basic Physical Properties of CH_4_ and H_2_

gas type	density (kg/m^3^)	LEL (%)	UEL (%)
CH_4_	0.6681	4.9	15.0
H_2_	0.0819	4.0	75.9

For multicomponent flammable mixture gas, the explosion limit can be calculated according to Le Chatelier’s law, as shown by eq 11.
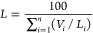
11Based on the existing HBNG applications, the maximum value of HBR does not exceed 30%. Therefore, HBRs selected in this work are 10, 20, and 30%. The corresponding LEL and UEL are calculated, as shown in Table 5.

**Table 5:** LEL and UEL for Different HBRs

no.	HBR (%)	LEL (%)	UEL (%)
1	10	4.79	16.30
2	20	4.69	17.86
3	30	4.60	19.74

#### Work Conditions

The leakage volume rate of HBNG in the leak hole is obtained by converting the CH_4_ release volume rate of 22.2 m^3^/h from Lowesmith’s experiment^24^ to 9.5 mm diameter. Then, the mass flow rates for different HBRs in the leakage hole are calculated according to gas densities provided in Table 4. Based on the analysis requirements, 25 numerical solution cases are conducted in this work. The information is displayed in Table 6. Case 0 is used to test mesh independence, turbulence model, and wall functions. Case 1–3 results are used to analyze the initial stage of leakage. Case 1–9 results are used to analyze the full process evolution behavior of gas accumulation and ventilation. Case 10–24 results are used to analyze the ventilation process after extending the gas accumulation time.

**Table 6:** Work Conditions for Each Case

scenario	HBR (%)	mass flow rate (kg/s)	solution time (s)	solution process
case 0	0	0.00404	0–120	test
case 1	10	0.00376	0–120	gas accumulation
case 2	20	0.00341	0–120	gas accumulation
case 3	30	0.00304	0–120	gas accumulation
case 4	10	0.00376	120–3600	gas accumulation
case 5	20	0.00341	120–3600	gas accumulation
case 6	30	0.00304	120–3600	gas accumulation
case 7	10	0.00376	3600–9000	ventilation
case 8	20	0.00341	3600–9000	ventilation
case 9	30	0.00304	3600–9000	ventilation
case 10	10	0.00376	3600–5400	gas accumulation
case 11	20	0.00341	3600–5400	gas accumulation
case 12	30	0.00304	3600–5400	gas accumulation
case 13	10	0.00376	3600–7200	gas accumulation
case 14	20	0.00341	3600–7200	gas accumulation
case 15	30	0.00304	3600–7200	gas accumulation
case 16	10	0.00376	3600–13 200	ventilation
case 17	20	0.00341	3600–13 200	ventilation
case 18	30	0.00304	3600–13 200	ventilation
case 19	10	0.00376	5400–15 000	ventilation
case 20	20	0.00341	5400–15 000	ventilation
case 21	30	0.00304	5400–15 000	ventilation
case 22	10	0.00376	7200–16 800	ventilation
case 23	20	0.00341	7200–16 800	ventilation
case 24	30	0.00304	7200–16 800	ventilation

#### Numerical Method

In this work, the finite volume method is applied to discrete the equations, and the PISO algorithm is applied to deal with the pressure–velocity coupling. Detailed information is shown in Table 7. For *t* = 0–120 s, the adaptive time step method is applied based on CFL < 2. The maximum time step does not exceed 0.5 s. For the remaining solution process, the fixed time step method is applied as 0.5 s. In the whole solution process, the maximum iteration per time step is set as 250. The residual criteria are set to 1 × 10^–4^ for continuity, *x*-velocity, *y*-velocity, *z*-velocity, turbulence parameters, and species and 1 × 10^–6^ for energy.

**Table 7:** Coupling and Discrete Scheme

item	information
pressure–velocity coupling method	PISO
gradient spatial discretization	least squares cell based
pressure spatial discretization	PRESTO!
energy spatial discretization	second-order upwind
momentum spatial discretization	second-order upwind
species spatial discretization	second-order upwind
turbulent dissipation rate spatial discretization	first-order upwind
turbulent kinetic energy spatial discretization	first-order upwind
transient formulation	bounded second-order implicit

#### Monitoring Points for Analysis

**Figure 5:**
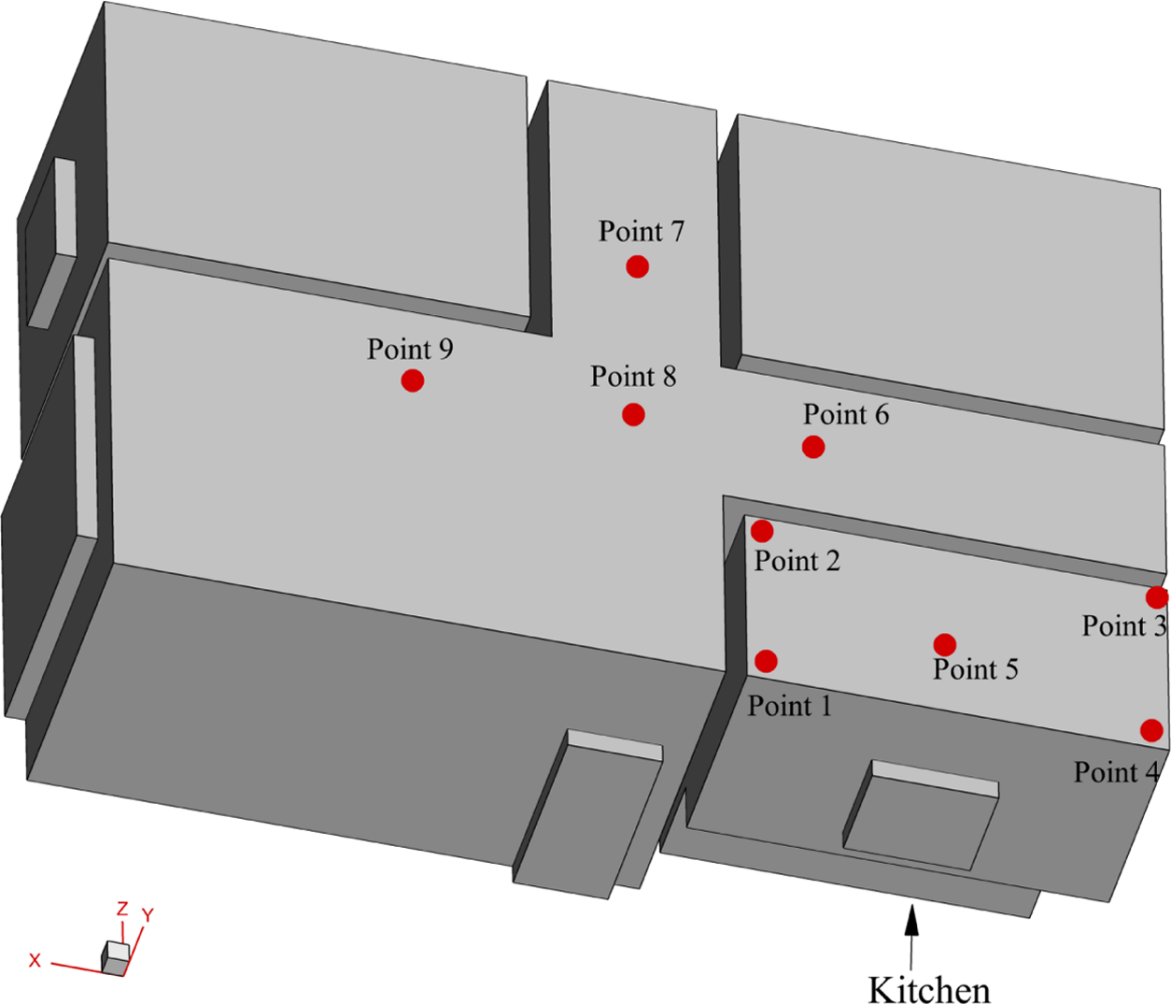
Layout of monitoring points.

Nine monitoring points are set near the house’s ceiling for analyzing the leakage process, as shown in Figure 5. The locations of the monitoring points are presented in Table 8 for reference.

**Table 8:** Monitoring Point Locations

no.	location (m)	no.	location (m)
point 1	(4,3,–7)	point 6	(3,3,–4)
point 2	(4,3,–5)	point 7	(5,3,–2)
point 3	(0,3,–5)	point 8	(5,3,–4)
point 4	(0,3,–7)	point 9	(8,3,–4)
point 5	(2,3,–6.75)		

### Model Validation

#### Mesh Independence Analysis

In order to select the appropriate mesh type and cell number for the simulation, different meshes are tested for case 0. All meshes are encrypted near the leak hole, and prismatic layers are generated near the walls. Mesh type and cell numbers are shown in Table 9.

**Table 9:** Different Mesh Information

mesh type	cell number	minimum size (mm)	maximum size (mm)
poly-hex	389 533	2	128
poly	333 713	2	128
hex-dominant	967 078	2	128
tetra	15 15 783	2	128
tetra-hex	1 183 627	2	128
poly-hex2	404 789	1	128
poly-hex3	841 258	1	64

**Figure 6:**
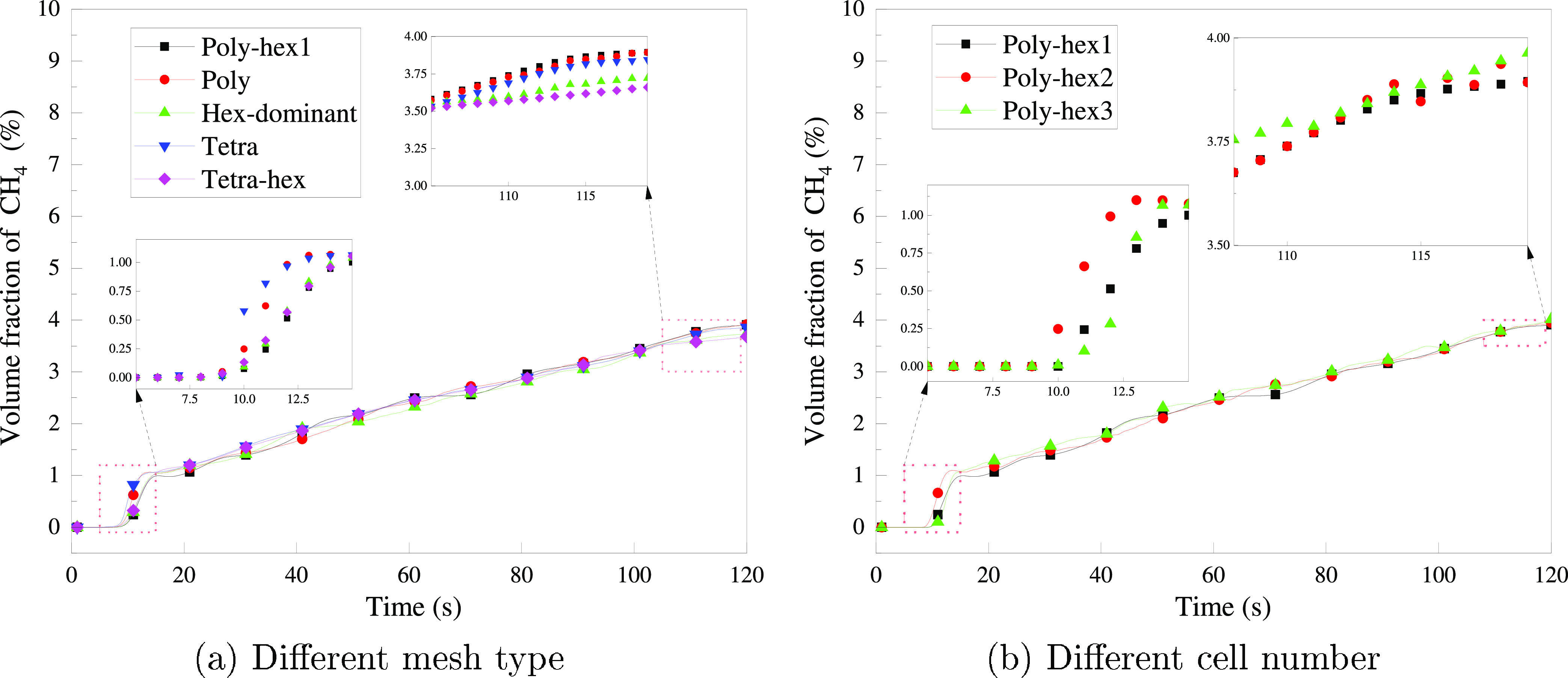
CH_4_ volume fraction variation at the monitoring point.

Figure 6(a) shows the variation of CH_4_ at a monitoring point in the kitchen for different mesh types. It shows that the effect of mesh type on the results is not significant. The maximum relative error after *t* = 60 s is not greater than 10%. Therefore, poly-hex mesh with a smaller cell number and faster convergence is chosen for the simulation.

**Figure 7:**
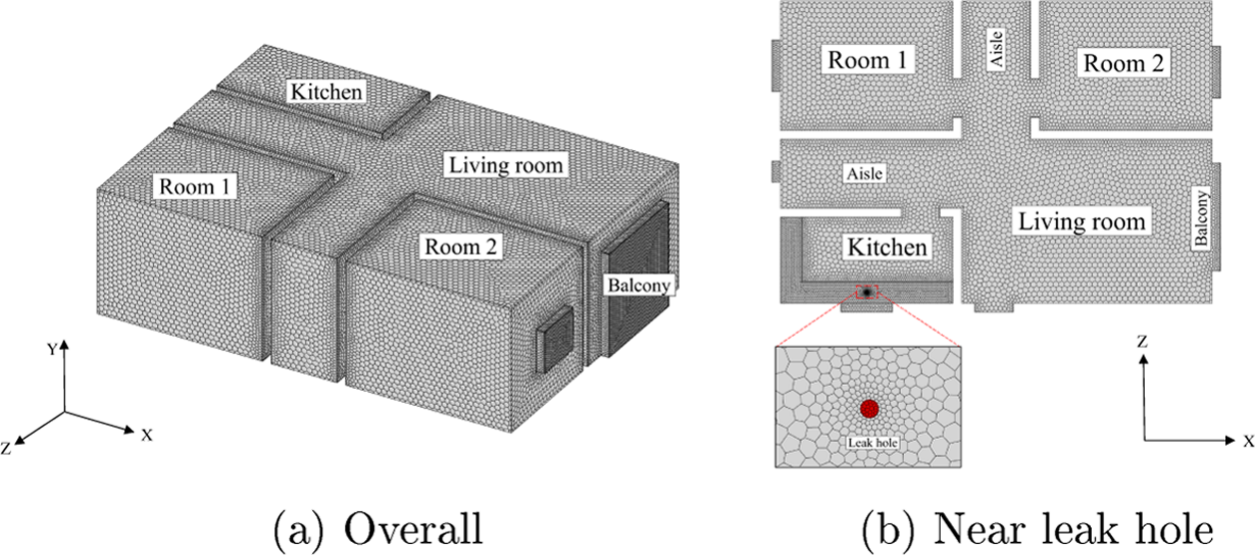
Schematic diagram of the generated mesh.

After selecting the mesh type, it is also necessary to select the appropriate cell number. Mesh ploy-hex2 and poly-hex3 are obtained by adjusting the maximum and minimum sizes shown in Table 9. The above comparison process is repeated, and the results are shown in Figure 6(b). It shows that the variation in the cell number has no significant effect on the simulation results and that the mesh independence has been verified. The final generated mesh is shown in Figure 7.

#### Numerical Simulation Method Validation

**Figure 8:**
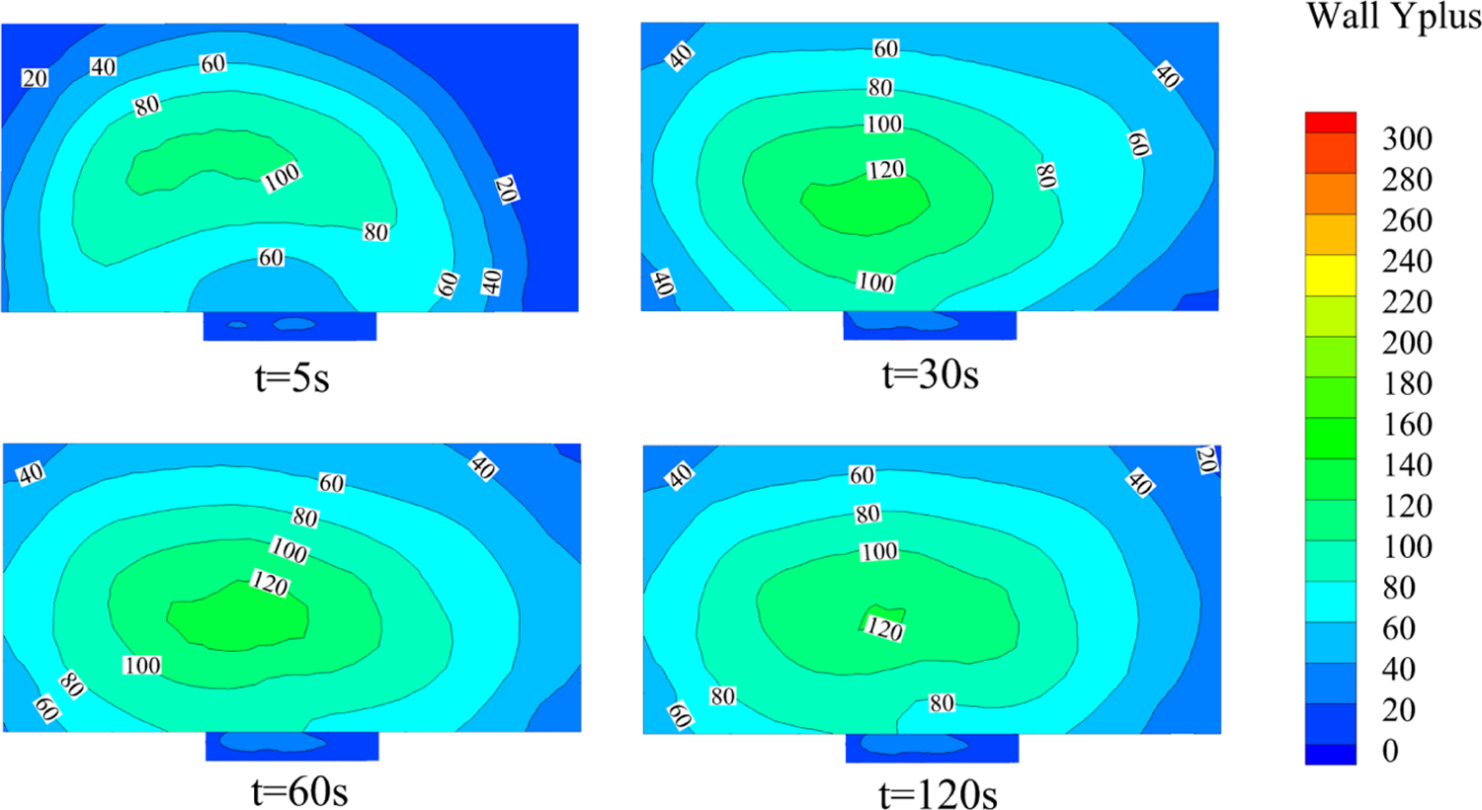
Y plus results in the critical area.

In order to determine the match between the scalable wall function and the near-wall mesh, case 0 is selected for testing. The critical area of flow is selected as the wall surface directly above the gas jet in the kitchen. Dimensionless wall distance Y plus results are shown in Figure 8. It is mainly distributed between 40 and 120. It indicates that the near-wall treatment method is reasonable.

**Figure 9:**
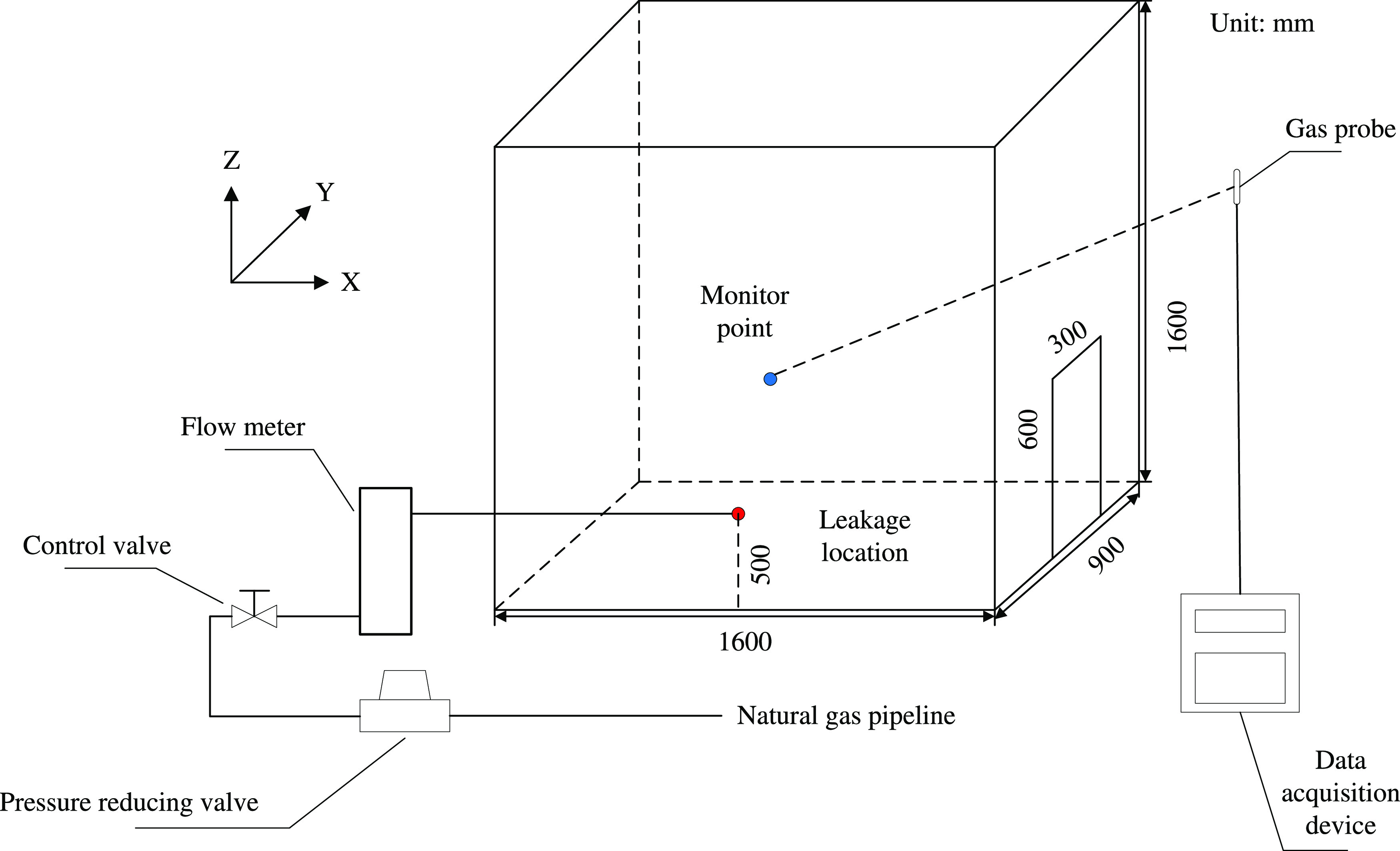
Model and experiment system in Huang’s research.

The gas leakage experiment conducted by Huang et al.^25^ is used to validate the reliability of the numerical simulation method. Huang’s experiment was conducted in a kitchen model. The model size and experimental system are shown in Figure 9. The leakage resource is the natural gas pipeline, and the pressure is reduced to 2000 Pa by a valve. The gas source pressure is essentially the same as in this work, and both can generate a gas jet. The flow meter and the control valve ensure that the pressure fluctuation is as low as enough. With the bottom left corner of the kitchen model as the coordinate origin, the leak location (800, 0, 500) and the monitoring point (800, 450, 800) are shown in Figure 9. The volume fraction of leaked gas at the monitor point is converted to a mass fraction as the reference for verification in this work. When leakage occurs, the CH_4_ volume fraction at the monitor point is collected every 60 s. It is sent to the data acquisition device via a gas probe.

**Figure 10:**
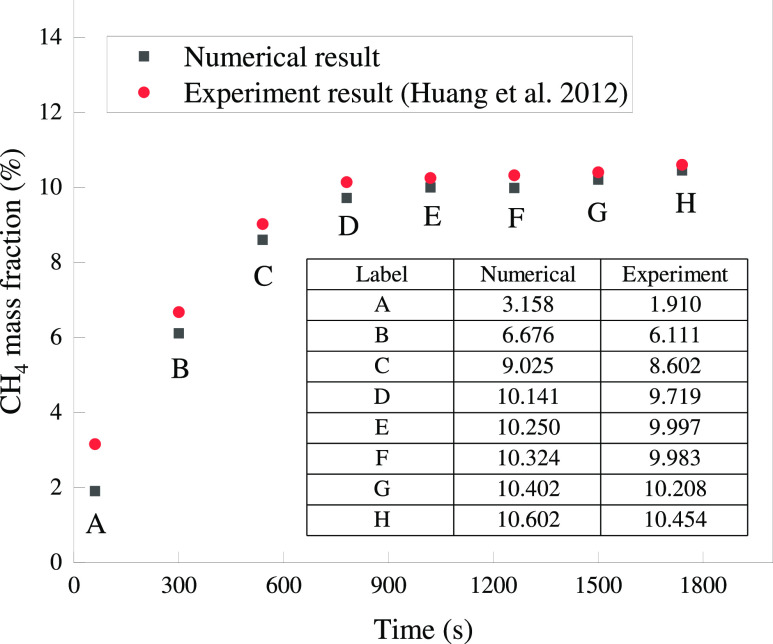
Data comparison of the simulation and the experiment by Huang et al.

The comparison results are shown in Figure 10. The simulated values are slightly larger than the experimental values all the time. The difference is more noticeable when *t* = 0–200 s. The difference in results at the initial stage may be caused by inconsistent release moments. The possibility that the initial direction of gas flow does not exactly coincide between the experiment and the numerical simulation is also a possible reason. The mean relative error at each point is 4.1%. Overall, the results of the experiment and the simulation in the near field are in high agreement. It indicates that the numerical method in this work is sufficiently reliable.

## Results and Discussion

### Analysis of the Gas Leakage Initial Stage

#### Gas Distribution at Different Heights (*t* = 0–120 s)

**Figure 11:**
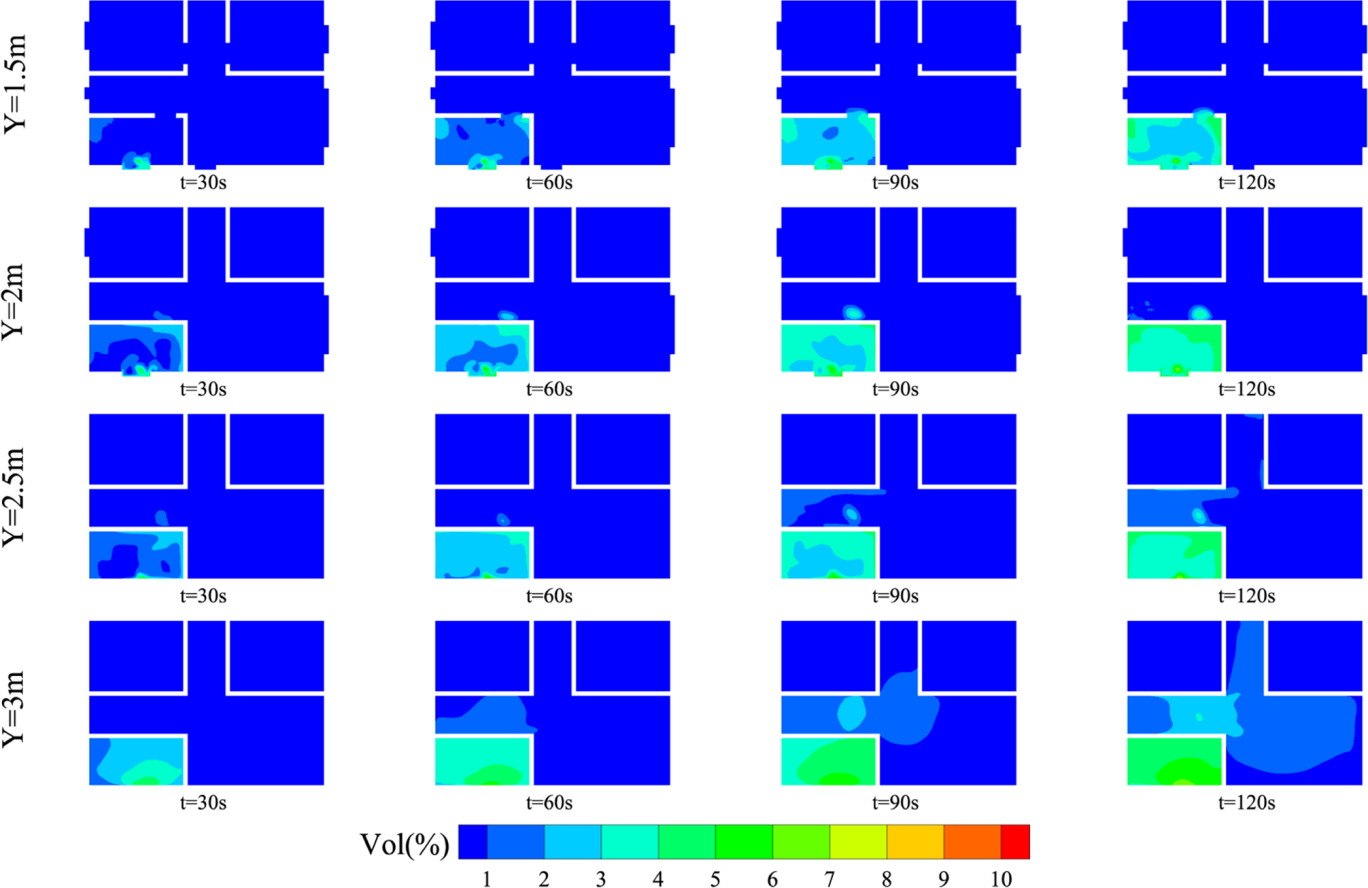
Leaked gas distribution at different heights (*Y* = 1.5, 2, 2.5, and 3 m).

**Figure 12:**
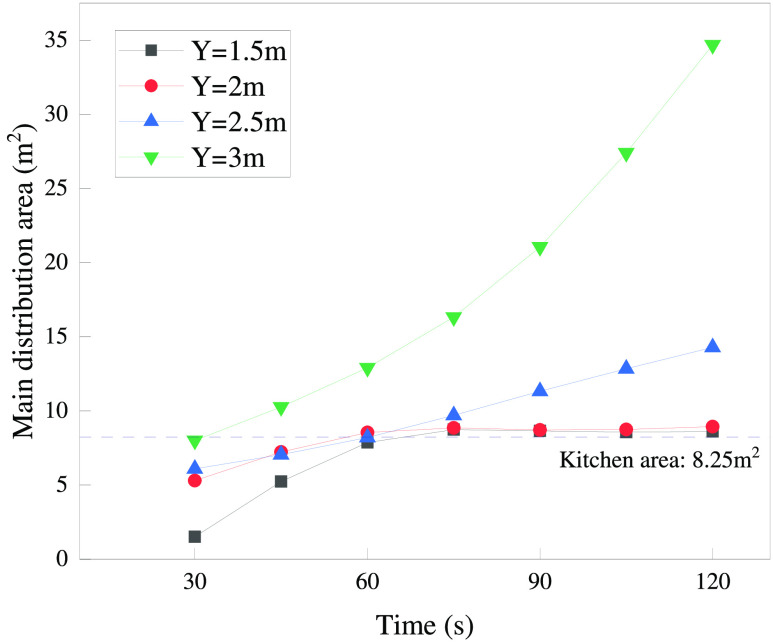
Leaked gas main distribution area at different heights.

The evolution of HBNG mixture distribution during the initial leakage stage (*t* = 0–120 s) is explored. In order to compare the layering properties of leaked gas at different heights, *Y* = 1.5 2, 2.5, and 3 m planes are selected to observe the evolution of CH_4_ and H_2_ mixture gas distribution. The results are shown in Figure 11. It can be found that the range of distribution varies significantly at different heights. In general, the range of gas distribution expands significantly with the evolution of time. At the same leakage time, the distribution range of leaked gas increases with increasing height. When *t* = 120 s, the difference in the distribution range is significant. The space where the leaked gas volume fraction is higher than 1% is considered as the main distribution area. At *Y* = 1.5 and 2 m, leakage gas is mainly distributed in the kitchen. The maximum volume fraction in the kitchen reaches 5%, and in the remaining space, it is hardly more than 1%. At the ceiling height (*Y* = 3 m), leaked gas has flowed into the living room and the aisle. The maximum volume fraction in the kitchen reaches 6%, which is not a significant increase from *Y* = 1.5 m. However, the volume fraction is higher than 1% in most areas of the living room and aisles. In the aisle close to the kitchen, the maximum value can exceed 3%. The main distribution areas under different heights are shown in Figure 12. The reference line indicates the kitchen area as 8.25 m^2^. It shows that the area at *Y* = 3 m is much larger than that at other heights. When *t* = 120 s, the values at *Y* = 1.5 and 2 m are only slightly higher than the kitchen area. When analyzed in combination with Figure 11, the excess portion is the portion of the aisle close to the kitchen door.

The distribution at different heights during the initial leakage stage reflects the characteristics of the gas flow. Under the effect of pressure differences, HBNG leakage is shown as a jet flow. The maximum gas velocity in this work is approximately 0.25 Ma. It is a subsonic flow, and the exit densimetric Froude number is used to describe the ratio of momentum and buoyancy effects.^26^ The calculation of *Fr* is shown in eq 12.
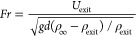
12

According to the analysis for eq 12, the initial jet velocity and the density difference between leaked gas and the external environment affect the *Fr* value. The buoyancy effect is significantly influenced by the difference in gas density. The momentum is reflected by the value of gas velocity. Therefore, *Fr* is able to reflect the differences in dominant influence on the gas jet. The criteria for judgment are shown in Table 10.

**Table 10:** Gas Jet Dominated Mechanisms under Different *Fr* Numbers

*Fr* range	mechanisms
*Fr* < 10	dominated by buoyancy
10 < *Fr* < 1000	dominated by momentum and buoyancy
*Fr* ≥ 1000	dominated by momentum

**Figure 13:**
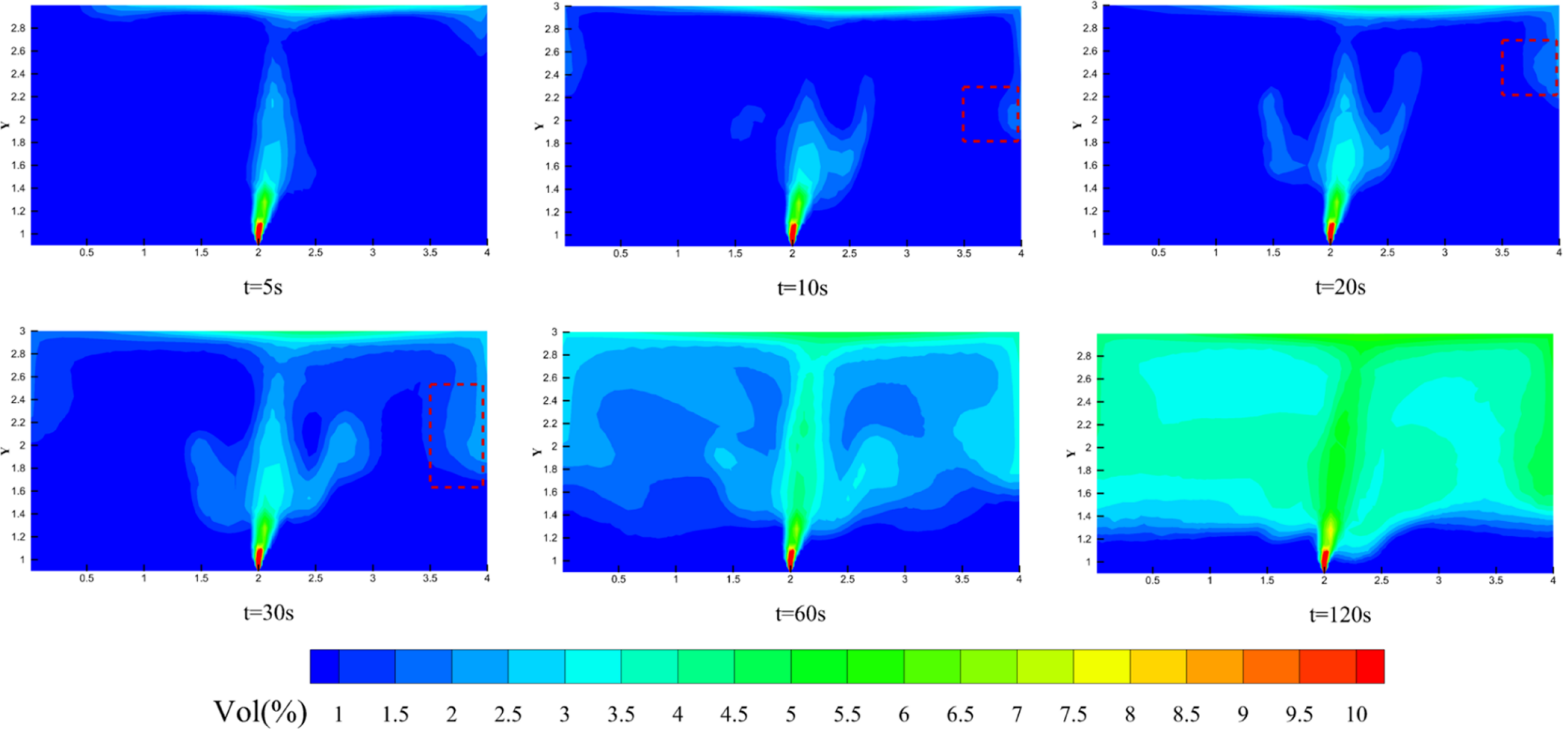
Evolution of gas distribution in the kitchen (*t* = 0–120 s).

**Figure 14:**
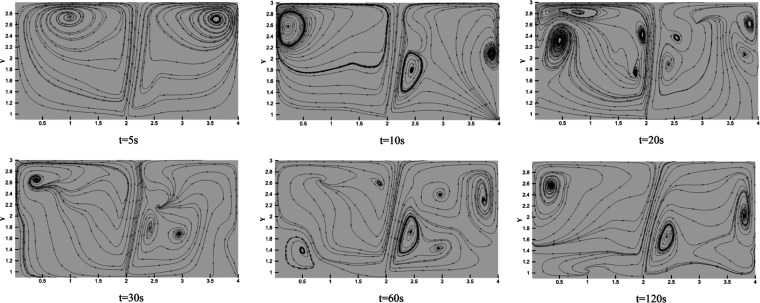
Evolution of streamlines in the kitchen (*t* = 0–120 s).

According to eq 12, *Fr* in this work is approximately 238 to 293. It is of the type dominated by momentum and buoyancy. The leaked gas moves toward the ceiling under the combined effect of initial momentum and buoyancy. The initial jet entrains the surrounding air, and momentum exchange takes place. The cross area of the jet increases with it. The gas concentration increases rapidly in the higher level of the kitchen, as shown at *t* = 10 and 20 s. Then, the front gas moves in a horizontal direction after it contacts the ceiling. The leaked gas flows along the wall gradually, consuming momentum under the effects of wall roughness and viscosity. It moves horizontally for a distance and then contacts the vertical wall. At this point, the gas flow front produces a return gas flow, as shown in the red frame of Figure 13. The streamlines also reflect the behavior of gas flow, as shown in Figure 14. The space at the top of the room is then further filled, gradually forming the HBNG gas cloud. As the velocity of the gas flow front decreases further, the leaked gas gradually mixes in the field far away from the leak location. At this point, it shows a degree of layering properties, as shown in Figure 13 at *t* = 120 s. The effect of buoyancy is significant in this field. The density of leaked HBNG in this work is much less than air, so leaked gas is still able to be distributed at high levels after the initial momentum has been nearly dissipated. It makes the HBNG gas cloud able to suspend at the top of the room without a strong convection effect.

Due to the obstructive effect of the walls, the leaked gas into the space beyond the kitchen is mainly driven by a concentration gradient. The concentration gradient is not sufficiently large at the initial leakage stage. For these reasons, the gas accumulates at high levels and flows slowly into the space beyond the kitchen.

**Figure 15:**
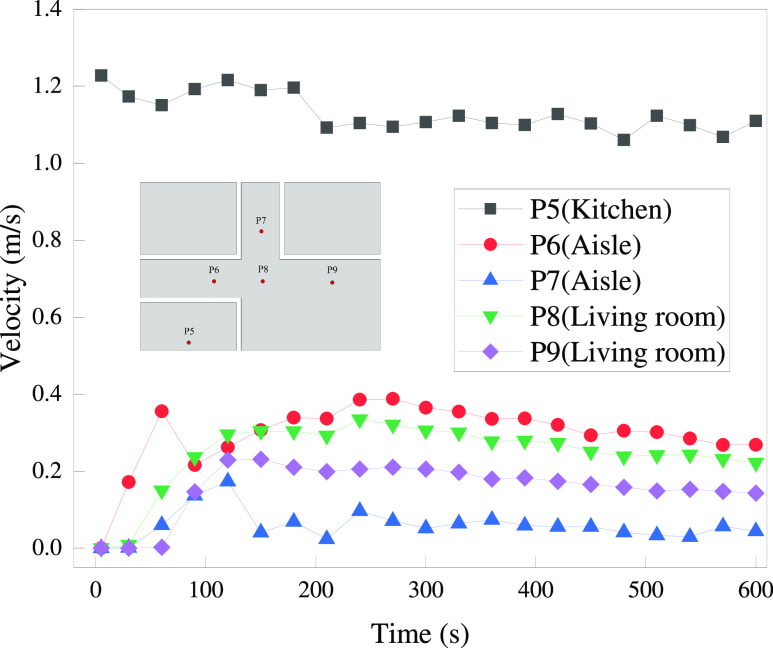
Velocity variation of points at *Y* = 3 m.

For the distribution of HBNG in the space beyond the kitchen, the main factors to be considered are the convective effect of the gas flow and the diffusion effect driven by the concentration difference. As the house is a closed space in this stage, there is no forced convective drive from the external environment. In order to assess the convective effect, monitoring points are placed at the top of the kitchen, living room, and aisle. The velocity variation at each monitoring point is shown in Figure 15. The initial velocity of the gas jet is significantly reduced after the viscosity and wall effects. The results illustrate that convection is significantly lower in the space beyond the kitchen than in it. Temperature differences between different spaces may produce natural gas convection. However, the temperatures of the environment and gas jet in this work are assumed to be the same according to the assumptions mentioned earlier. It can be considered that the natural convection induced by the temperature difference is insignificant. The significant layering of leaked gas in Figure 13 also confirms that the convection effect is not apparent far away from the leak location. At this point, the development of the leaked gas cloud is driven more by the concentration gradient. In the lack of sufficient driving forces, the expansion of the leaked gas distribution range is significantly slower than that in the kitchen.

The kitchens in this work that separate the kitchen from the rest of the living space with doors and walls are called closed kitchens. According to the evolution of leaked gas, household gas alarms should be installed at a high level in such enclosed kitchens. Due to the possible delay in ignition for gas, false alarms may occur when installed directly above the stovetop. It is more appropriate to install household gas alarms at a horizontal distance from the stove, as required by GB/T34004-2017.^27^ The lower density of HBNG than air makes it probable that gas clouds form at high levels. Even if the door is not closed, the obstructive effect of walls at high levels can slow down the expansion of gas distribution. Compared to popular open kitchens without dividing walls and doors, it can be considered that closed kitchens lose some space utilization in exchange for safety.

#### Gas Distribution for Different HBRs (*t* = 0–120 s)

**Figure 16:**
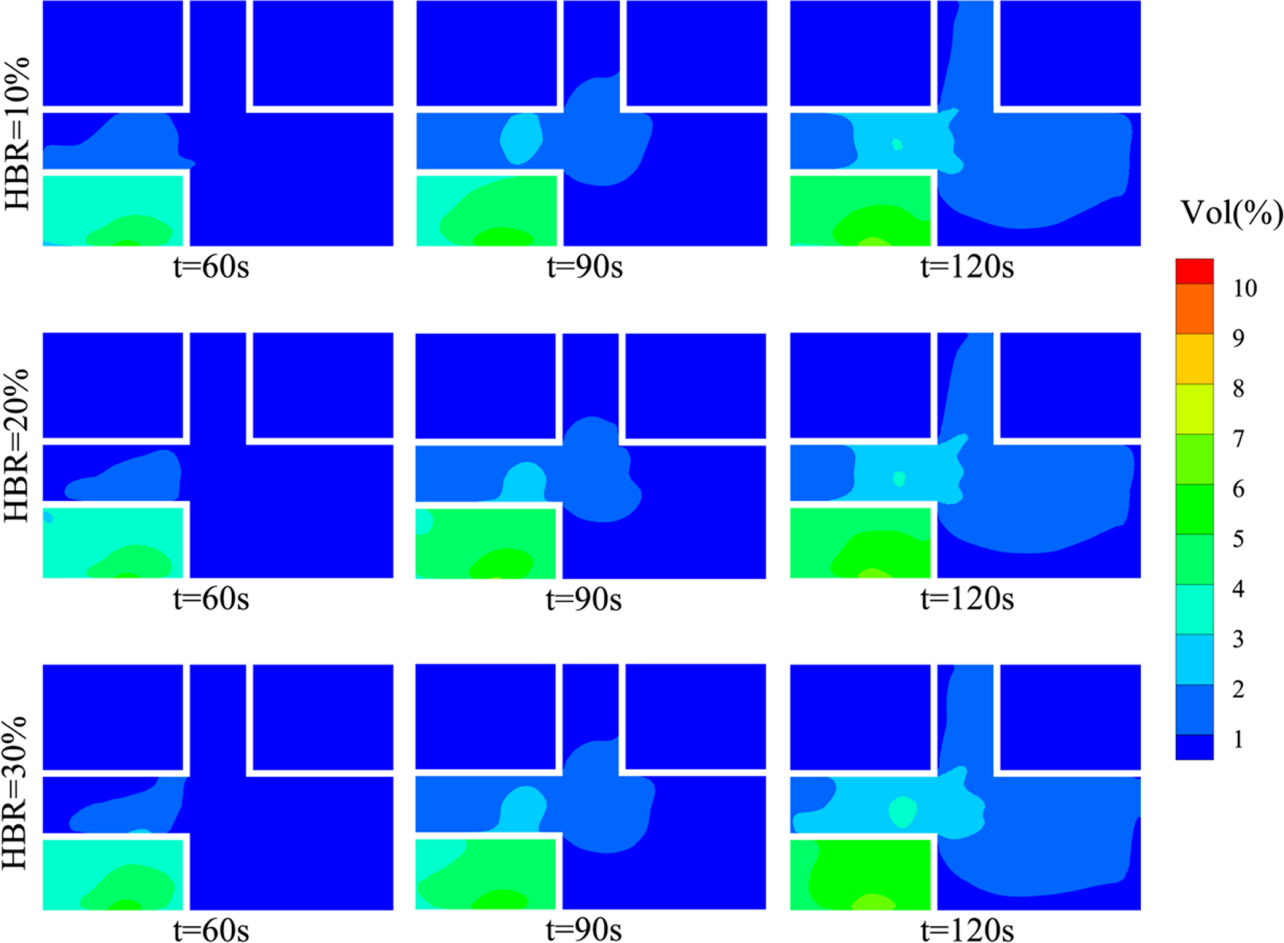
Gas distribution for different HBRs at *Y* = 3 m.

In this section, the effect of HBR on the leaked gas distribution is investigated. The leaked gas distribution for different HBRs at *Y* = 3 m is shown in Figure 16. It can be found that the distribution range of the leaked gas does not vary significantly with HBR at the same time. It is possible that HBR is not chosen to large values in this work and that the differences in properties between the gas mixtures are not significant enough. Therefore, there are no significant differences in the range of gas distribution reflected.

**Figure 17:**
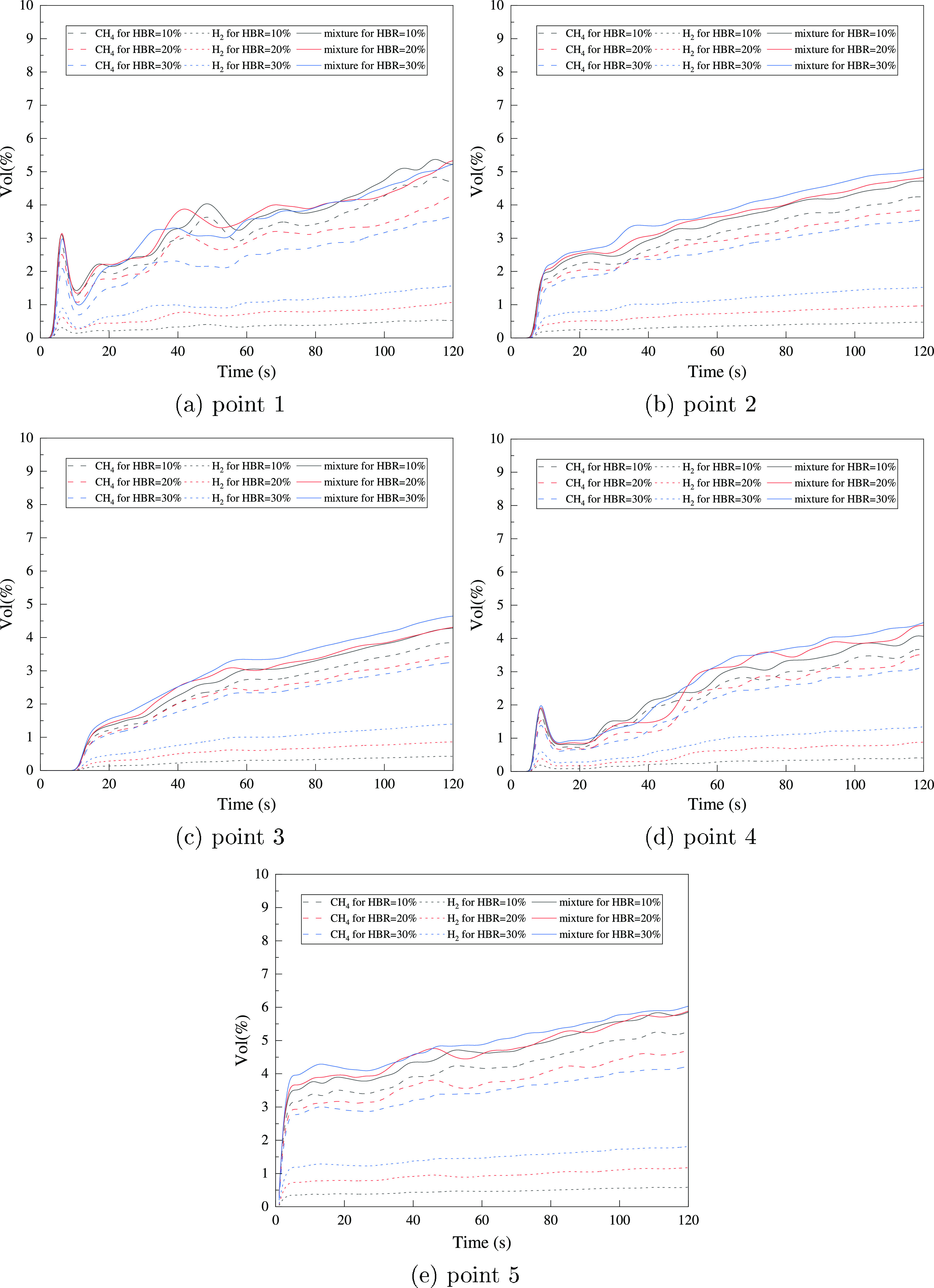
Gas distribution for different HBR values at monitoring points.

For analysis on smaller time scales, monitoring points are set up at the ceiling above the leak location to analyze the evolution of the gas distribution. The HBNG mixture, CH_4_and H_2_, at every monitoring point varies, as shown in Figure 17. It can be found that the volume fractions of CH_4_ and H_2_ vary significantly with HBR, but their sum shows consistency.

**Figure 18:**
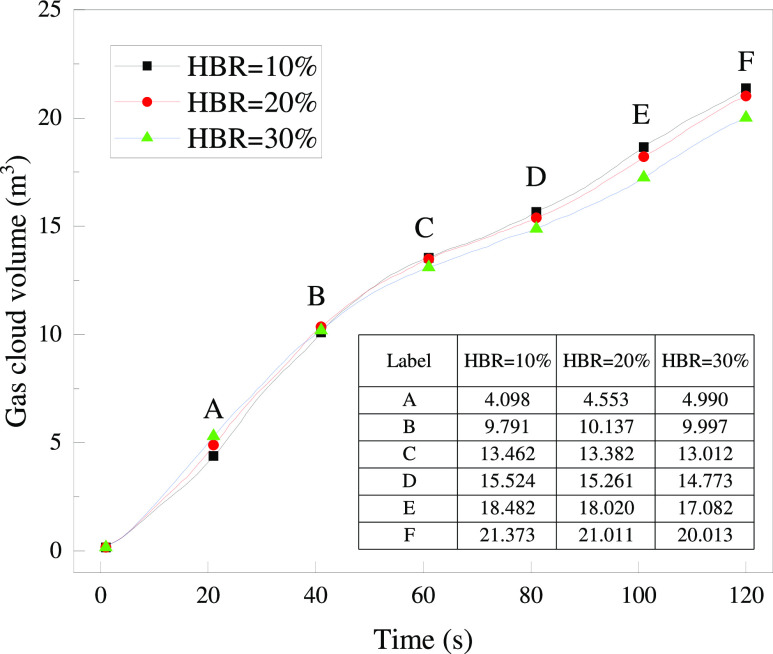
Variation of the gas cloud volume for different HBRs (*t* = 0–120 s).

Differences in gas distribution for different HBRs can also be analyzed by the gas cloud volume. According to the standard GB 55009-2021,^28^ the alarm concentration limit for household combustible gas detectors should not exceed 25% LEL. In this work, the area where the detector can alarm is considered as the gas cloud of HBNG. The variation curve of gas cloud volume is shown in Figure 18 by selecting the LEL according to Table 5. It can be found that the difference in the volume of gas clouds for different HBRs is not significant in general. Due to the close LELs of CH_4_ and H_2_, the LELs of HBNG for different HBRs do not exceed 0.3%. It leads to little difference in the gas cloud volume. A certain difference is gradually shown with increasing tendency when *t* = 46–120 s. The volume for a larger HBR is slightly lower. But the overall maximum difference is 1.3 m^3^, less than 1% of the house volume (198.5 m^3^). It is difficult to determine whether the difference at *t* = 60–120 s is due to different HBRs. These differences may be the result of cumulative errors in the simulations, and more microscale studies are needed to determine this. In summary, the HBR does not have a significant effect on the distribution of leaked gas in the initial leakage stage.

### Analysis of the Gas Accumulation and Ventilation Processes

In this part, the analysis is conducted on a longer time scale. Some time after the leakage occurs, the gas in the house forms a hazardous gas cloud that reaches the explosion limit. The hazardous gas cloud is defined as the area where gas concentrations meet the LEL and UEL range in Table 5. The volume of the hazardous gas cloud is considered as the sum of volumes of all cells’ central values meeting the LEL and UEL range.

#### Gas Accumulation Process (*t* = 0–3600 s)

**Figure 19:**
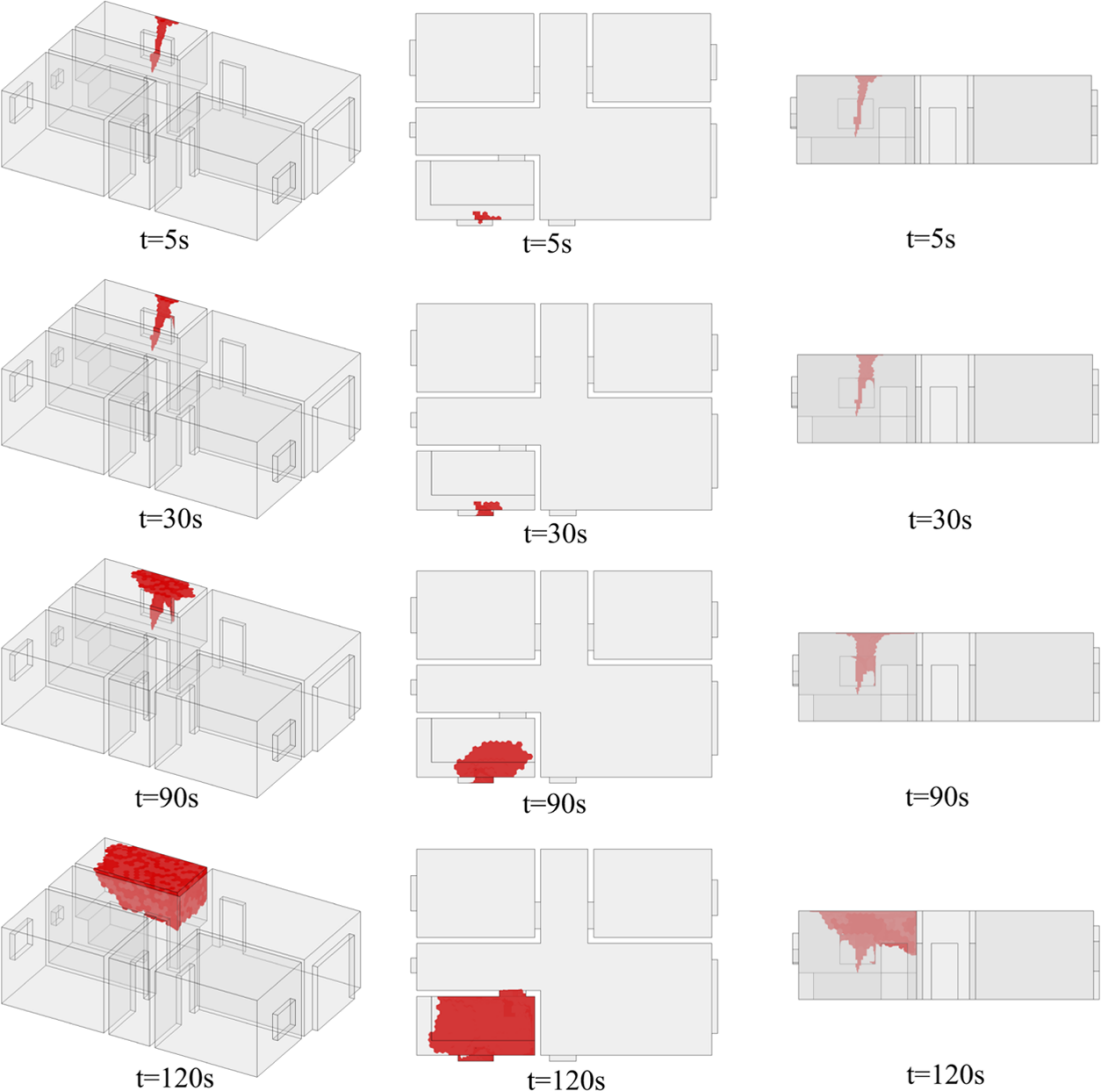
Variation of the hazardous gas cloud (*t* = 0–120 s).

**Figure 20:**
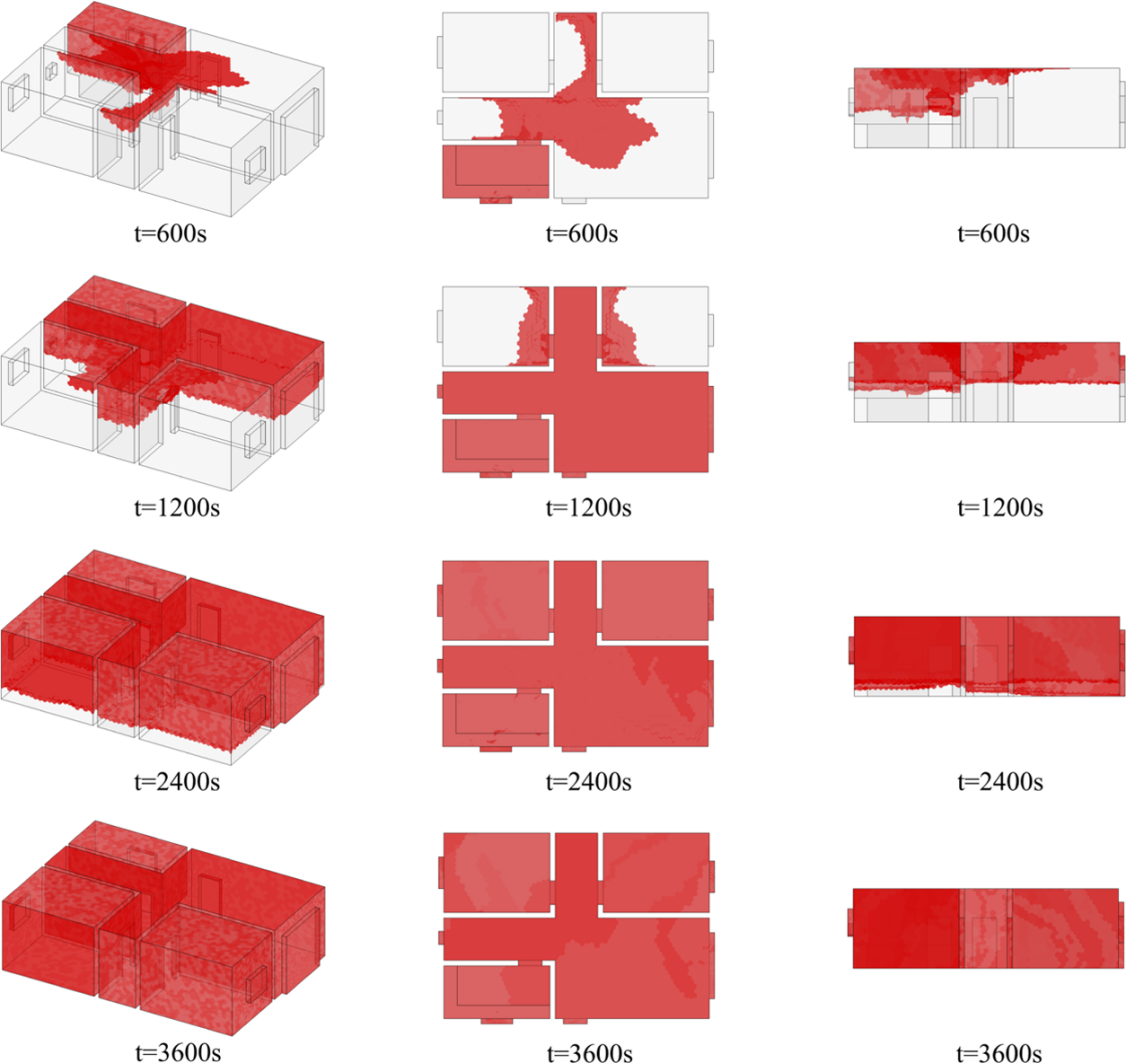
Variation of the hazardous gas cloud (*t* = 600–3600 s).

The evolution of the hazardous gas cloud at *t* = 0–120 s and *t* = 600–3600 s is shown in Figures 19 and 20. Corresponding to the distribution in Figure 11, the hazardous gas cloud is first generated at high levels in the kitchen. At *t* = 120 s, the hazardous gas cloud is already evident in the kitchen, which is mainly distributed above the height of the door frame. Due to the asymmetrical ventilation conditions in the kitchen, the hazardous gas cloud is shaped irregularly.

**Figure 21:**
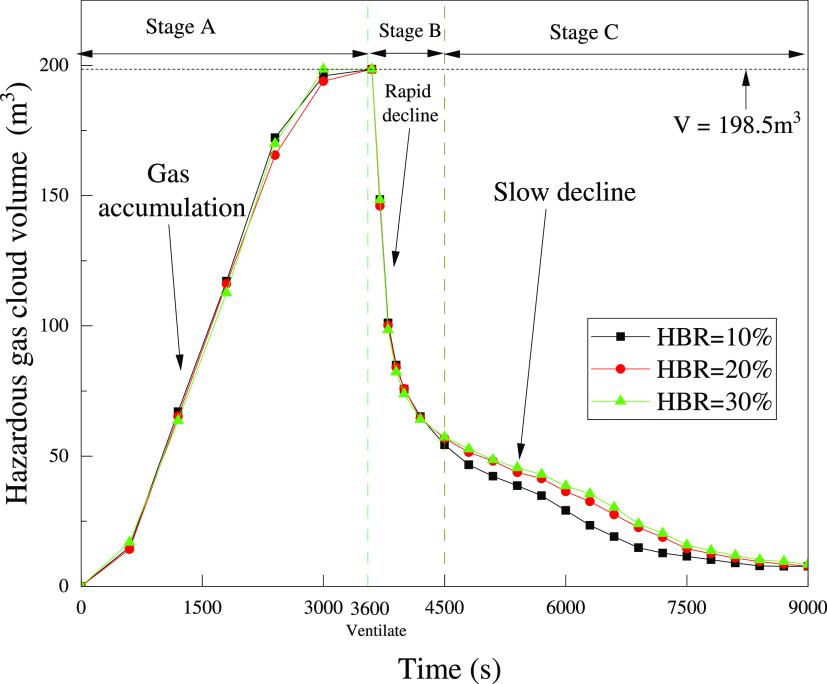
Variation of the hazardous gas cloud volume in the whole process.

Afterward, the hazardous gas cloud continues to develop into other spaces. At *t* = 600 s, the hazardous gas cloud appears in the high levels of the aisle and living room. At *t* = 1200 s, it appears in room 1 and room 2 near the door. At *t* = 2400 s, the volume of the hazardous gas cloud exceeds 75% of the interior volume of the house. At *t* = 3600 s, the hazardous gas cloud fills the entire house. Stage A in Figure 21 illustrates the whole process of gas accumulation. The final volume reaches 198.5 m^3^ which is the interior volume value of the house. The variation curves for different HBRs almost overlap. It indicates that the variation in the hazardous gas cloud volume for different HBRs is minimal in stage A. The formation behaviors of hazardous gas clouds are approximately the same for different HBRs. It validates the results in the previous section that HBR has no significant effect on gas distribution and evolution.

#### Ventilation Process (*t* = 3600–9000 s)

At *t* = 3600 s, all doors and windows are opened for ventilation to simulate the emergency response after a leakage accident has been found. In this process, it is assumed that the gas source is not cut off by the residents because of some difficulties.

**Figure 22:**
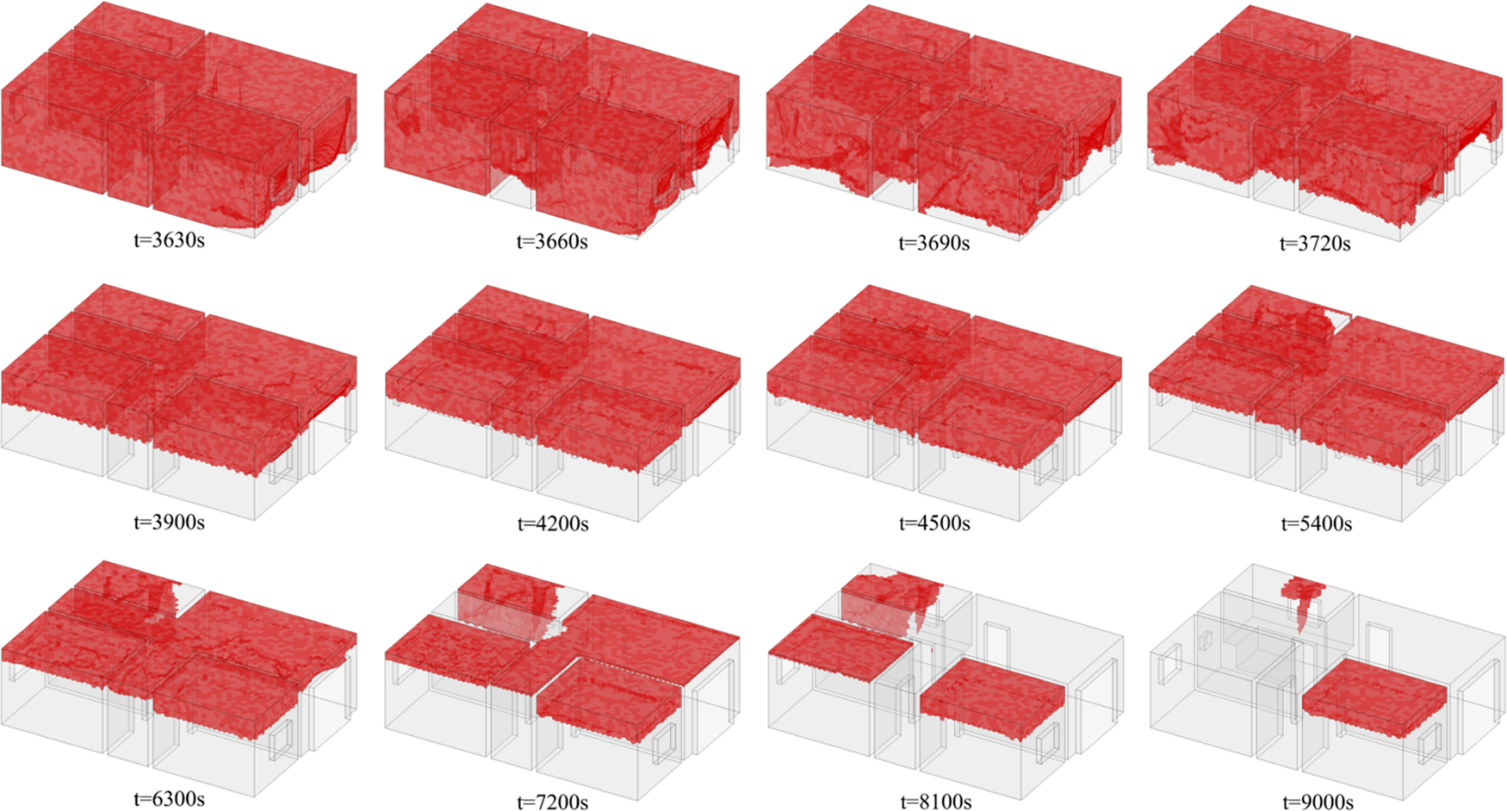
Variation of the hazardous gas cloud (*t* = 3600–9000 s).

The evolution of the hazardous gas cloud during *t* = 3600–9000 s is shown in Figure 22. It can be seen that the reduction of hazardous gas cloud volume is significant in the initial ventilation stage (*t* = 3600–4500 s) and then gradually slows down. At *t* = 8100 s, the gas cloud is reduced to room 1, room 2, and kitchen. At *t* = 9000 s, only a small portion of the hazardous gas cloud remains in the kitchen and room 2. The kitchen is the location of the gas source, so only gas cloud in room 2 is difficult to vent.

The variation in hazardous gas cloud volume is shown as stages B and C in Figure 21. The two stages of rapid (*t* = 3600–4500 s) and slow (*t* = 4500–9000 s) decline in volume are represented. The rapid decline in the hazardous gas cloud volume initially is due to diffusion driven by the difference in gas concentration between the interior and exterior. The balcony, windows, and doors have a large ventilation area and little obstruction to gas diffusion, which leads to a rapid diffusion from the lower levels of the house to the outside. When the gas cloud reduces to the upper part of the house, the diffusion path is obstructed by the walls. It leads to a reduction in the volume decline rate. Gas clouds in rooms 1 and 2 are slow to dissipate because of the heavy obstruction. Among them, the gas cloud in room 2 is not dissipated until *t* = 9000 s. The main reason is that room 2 does not have a balcony and has only one window. It leads to the worst ventilation condition. In this situation, it is necessary to apply convection as a momentum to dissipate the gas cloud.

**Figure 23:**
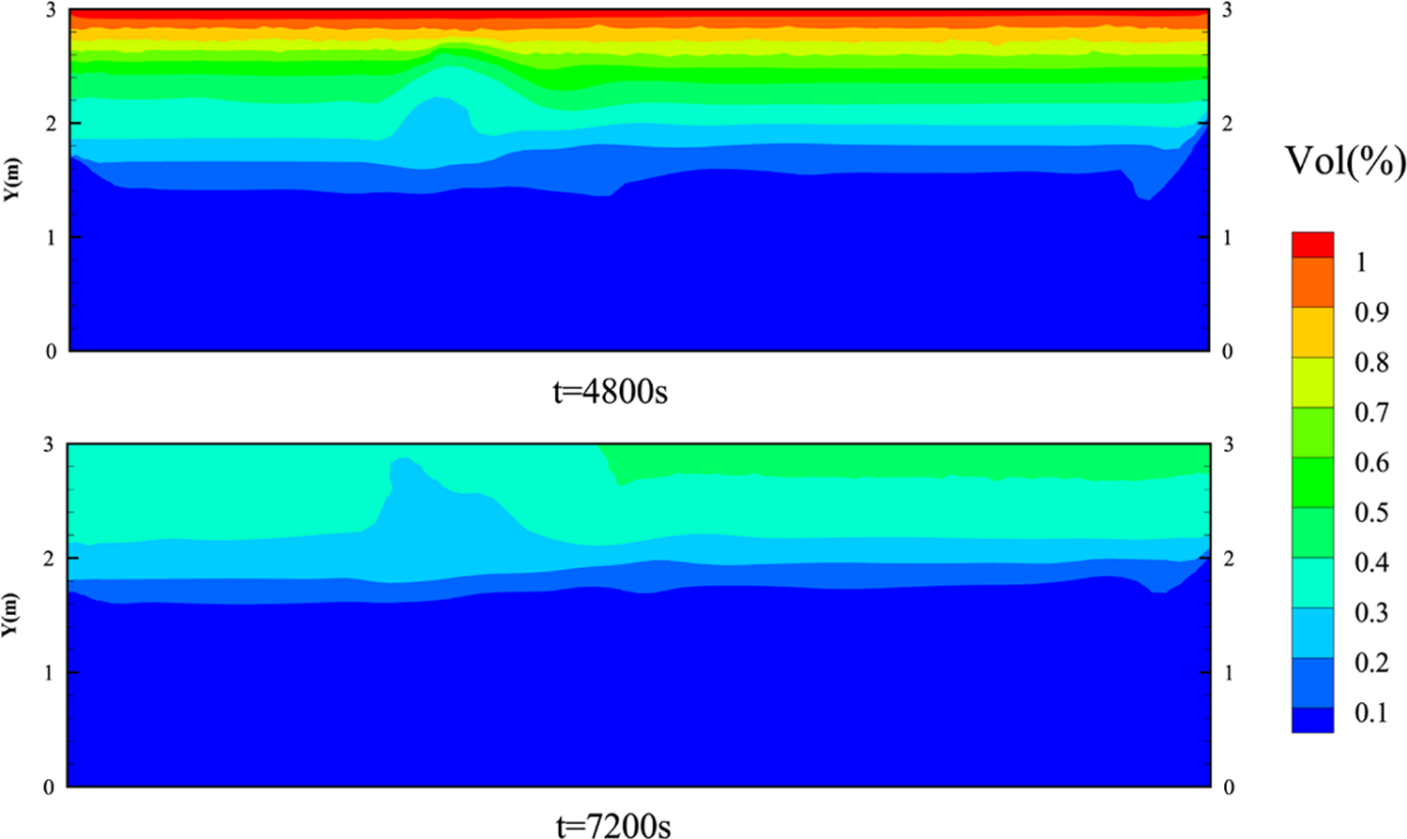
Layering properties of H_2_ in the vertical direction at the *XY* plane of the living room.

From Figure 21, it is found that the volume of the hazardous gas cloud for different HBRs starts to show a difference gradually after *t* = 4500 s. The decline in volume is more significant for a smaller HBR. The difference may be caused by the layering properties of the gas. The gas components show layering due to the combined effect of insignificant convection and the difference in density between H_2_ and CH_4_. As shown in Figure 23, the layering properties for H_2_ are significant after *t* = 4500 s. The concentration of H_2_ at the ceiling is higher and shows a uniform concentration gradient. The diffusion path at higher levels is obstructed by walls. Gas clouds with a high HBR have a higher proportion of H_2_ at the ceiling. So, the CH_4_ proportion is higher at the lower levels under the influence of gravity, making CH_4_ diffuse more easily to the outside. H_2_ at high levels is difficult to flow without a convective effect. Finally, it causes the gas cloud volume with higher HBR to decline slightly more slowly in Figure 21.

**Figure 24:**
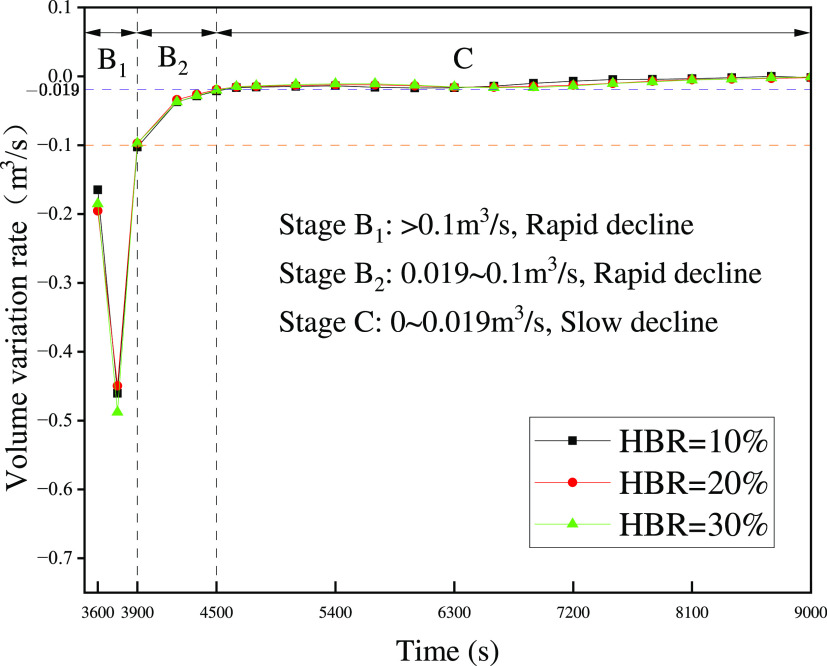
Volume variation rate of hazardous gas clouds in the ventilation process (*t* = 3600–9000 s).

The results for the volume variation rate with time are obtained by taking the first-order derivative of Figure 21. The more detailed division of the volume decline process is shown in Figure 24. The rapidly declining stage B (*t* = 3600–4500 s) is divided into stages B_1_(*t* = 3600–3900 s) and B_2_(*t* = 3900–4500 s). As the gas cloud volume declines, the volume variation rate becomes negative. For convenience, the absolute value of the volume variation rate is used here for analysis.

In stage B_1_, the volume variation rate is greater than 0.1 m^3^/s. The variation rate increases sharply and then declines sharply. The rapid variation at the beginning is due to the large ventilation area directly through the balcony, doors, and windows, which are in contact with the outside. The maximum variation rate is close to 0.5 m^3^/*s*. As the hazardous gas cloud volume declines, the ventilation area in contact with the gas cloud is reduced. The volume variation rate gradually declines.

In stage B_2_, the curve becomes progressively flatter, and the variation rate gradually decreases from 0.1 to 0.019 m^3^/s. It is evident that *t* = 3900 s is a clear transition point. In combination with Figure 22, *t* = 3600 s corresponds to the moment when the hazardous gas cloud is reduced to the upper edge of the window. After *t* = 3600 s, the gas diffusion starts to be obstructed by the walls at a high level. It leads to a significant transition in the volume variation rate. In stage C, the volume variation rate is already less than 0.019 m^3^/s and close to 0 m^3^/s. It leads to the variation of hazardous gas cloud shape needing a larger time interval to be observed. In stage C, the curves generally overlap and do not reflect the difference in Figure 22. The reason is that the values of variation rates differ but are too close.

### Analysis of the Ventilation Process for Extending Gas Accumulation Time

According to the previous results, it can be seen that at approximately 3600 s, the hazardous gas cloud can fill the whole house. If the ventilation time is postponed backward, it might affect the ventilation process to a certain extent. In the following, the ventilation process is investigated after extending the length of gas accumulation time.

**Figure 25:**
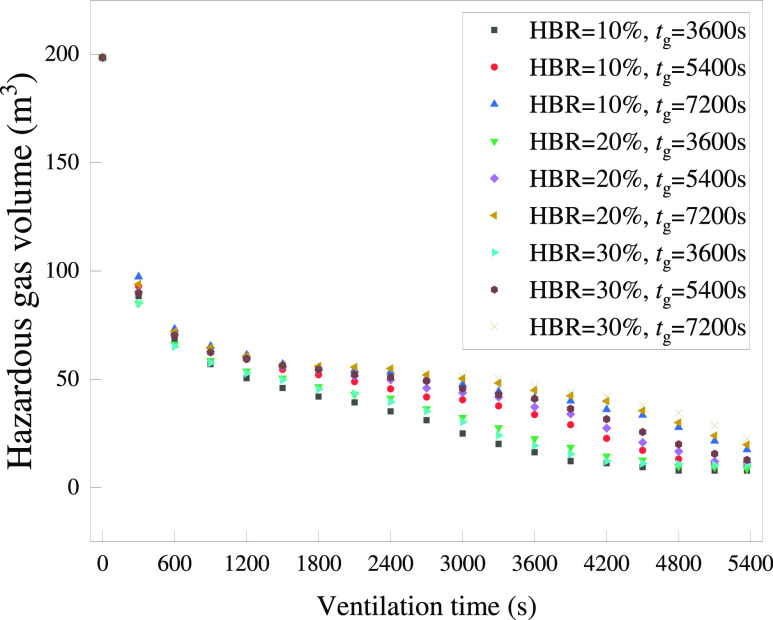
Variation of the hazardous gas cloud volume for different *t*_g_ and HBRs.

**Figure 26:**
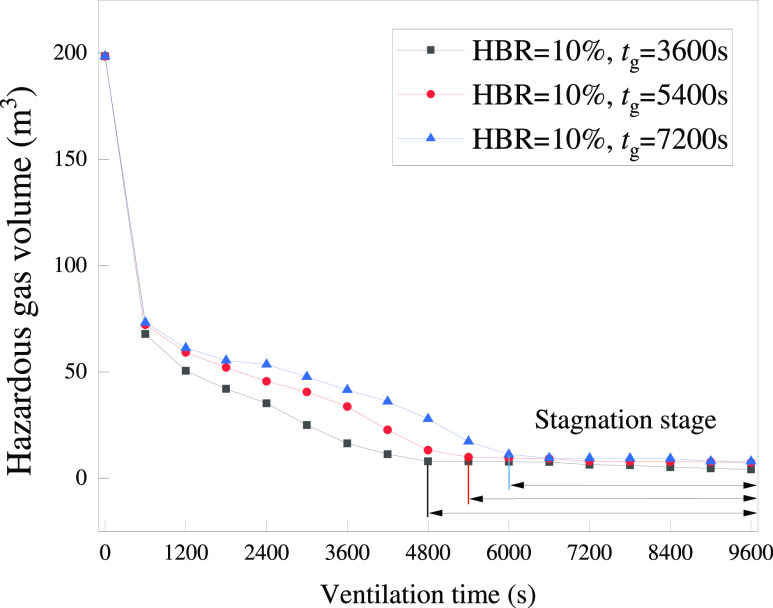
Variation of the hazardous gas cloud volume for different *t*_g_ and HBR = 10%.

**Figure 27:**
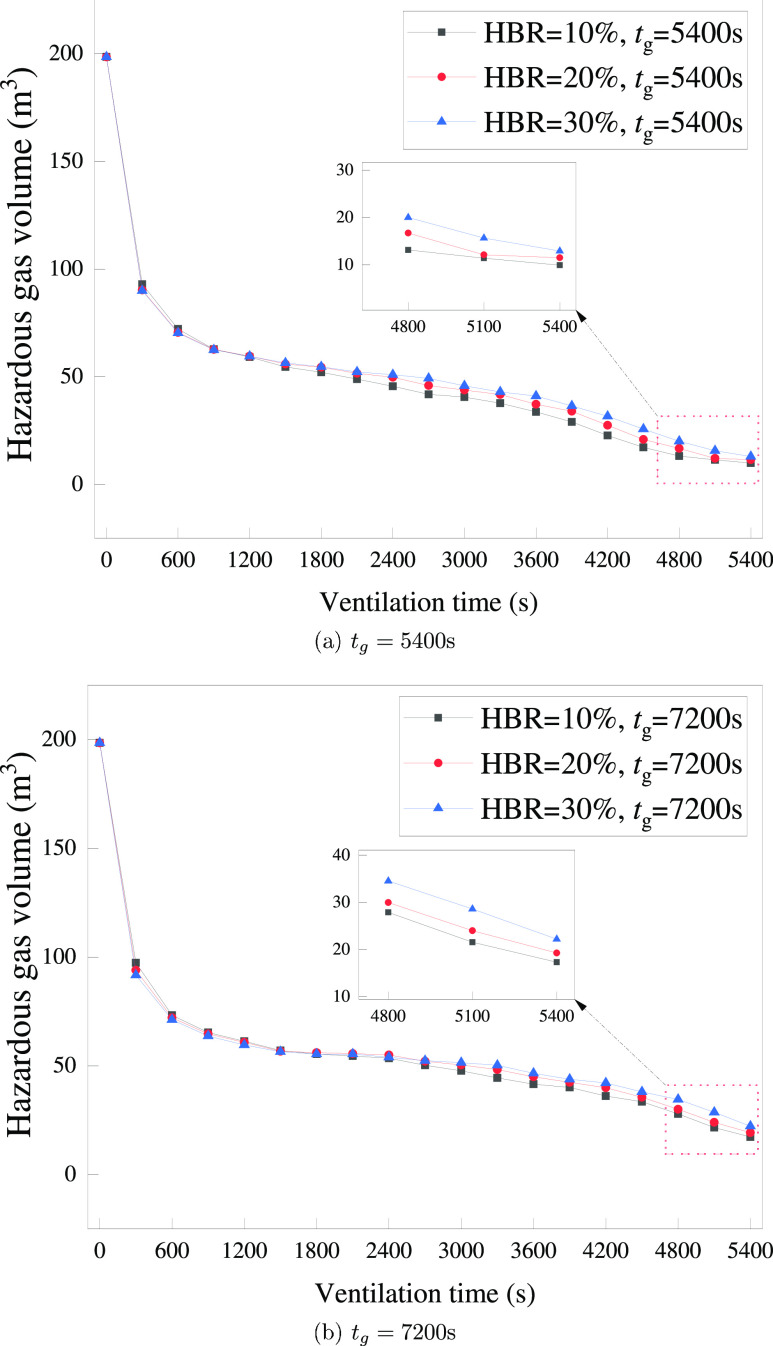
Variation of the hazardous gas cloud volume for different HBRs.

The gas accumulation time is set as *t*_g_, and the ventilation time is set as *t*_v_. *t*_g_ is extended from 3600 to 5400 and 7200 s. The variation of the hazardous gas cloud volume with time after the doors and windows were opened is shown in Figure 25. It is noticeable that the *X*-axis is selected for ventilation time *t*_g_ rather than the previous solution time *t*. *t*_g_ = 0 s represents the start of the ventilation process. From Figure 25, it is generally reflected that the larger the HBR and *t*_g_, the larger the hazardous gas cloud volume at the same moment. However, the data are too crowded to facilitate further analysis. Therefore, parts of the data have been extracted to produce Figures 26 and 27.

The variation in the hazardous gas cloud volume for HBR = 10% after extending *t*_g_ is shown in Figure 26. At *t*_v_ = 300 s, the gas cloud volume starts to show some difference. After that, the difference becomes more significant. After *t*_v_ = 4800 s, the curve of *t*_g_ = 3600 s is already close to being horizontal. The curves of *t*_g_ = 5400 and 7200 s are still significantly inclined. While continuing to ventilate, the curves of *t*_g_ = 5400 and 7200 s are close to horizontal after approximately *t*_v_ = 5400 and 6000 s. The horizontal values represent that the hazardous gas cloud is difficult to dissipate. Therefore, the horizontal section of the curves can be defined as the stagnation stage in the ventilation process. Under the lack of convection conditions, the ventilation process finally reaches a stagnant stage. Extending *t*_g_ significantly postpones the time point to the stagnation stage. During this stage, additional forced convection is required to reduce the hazardous gas cloud volume further. Figure 26 illustrates, in general, that *t*_v_ to reach the stagnation stage increases as *t*_g_ increases.

The variation in hazardous gas cloud volume for different HBR values after extending *t*_g_ is shown in Figure 27. It can be found that the general characteristics of the volume variation for *t*_g_ = 5400 and 7200 s are basically consistent. The volume difference is not significant during the rapid decline stage. After entering the slow decline stage, the higher HBR has a slightly larger gas cloud volume at the same moment. When *t*_v_ = 5400 s, it still reflects that the higher the HBR, the larger the hazardous gas volume that is difficult to dissipate, which is consistent with the previous finding for *t*_g_ = 3600 s.

## Conclusions

This paper investigates the gas accumulation and ventilation processes after HBNG leakage in a domestic house through CFD numerical simulations. In the initial leakage stage (*t* = 0–120 s), the effects of different heights and HBRs on the gas distribution are discussed. With the HBNG volume fraction greater than LEL as the hazardous gas cloud standard, the hazardous gas cloud volume evolution is analyzed in gas accumulation (*t* = 0–3600 s) and ventilation processes (*t* = 3600–9000 s). After the hazardous gas cloud filled the entire house, the effect of extending gas accumulation time on the ventilation process has also been analyzed. Based on the above results and discussion, the main conclusions are summarized as follows:

(1) In the initial leakage stage, the area of gas distribution is much larger at higher levels than that at lower levels. The leaked gas accumulates rapidly at high levels in the kitchen under the effects of buoyancy and initial momentum. The return flow caused by contact with the walls allows leaked gas to gradually mix with air and form a gas cloud at high levels. Due to the significant difference in density, the gas cloud is then able to remain suspended at high levels. At high levels in the house, the gas velocity in all other spaces is much less than that in the kitchen. When the gas cloud moves into the space beyond the kitchen, the lack of convection makes the expansion of the gas distribution range more driven by the concentration gradient.
(2) In the initial leakage stage, variations in HBR do not cause significant variation in the distribution range of leaked gas at the same time. Gas volume fractions at monitoring points are analyzed. The volume fractions of CH_4_ and H_2_ vary significantly at monitoring points for different HBRs, but their sum shows consistency. For the HBR in this paper (≤30%), the difference in gas properties led by different HBRs does not have a significant effect on HBNG distribution. In the same time range, the difference in gas cloud volume detected for different HBRs does not exceed 1% of the house’s interior volume. This validates that the effect of HBR on gas distribution is not significant.
(3) In the gas accumulation process, the hazardous gas cloud starts in the kitchen and gradually develops to other spaces. It starts from high levels and then develops to low levels. It fills the entire house at around *t* = 3600 s. The hazardous gas cloud volume variation curves for different HBRs are close to overlapping, which validates the conclusions in (2).
(4) In the ventilation process, the volume of the hazardous gas cloud declines rapidly at first and then slowly. The ventilation process can be divided into a rapid decline stage (*t* = 3600–4500 s) and a slow decline stage (*t* = 4500–9000 s) according to the volume variation rate. The rapid decline stage can be divided in detail according to the volume variation rate of 0.1 m^3^/s. The difference in the volume variation rate is mainly caused by the ventilation conditions. Gas clouds at lower levels are directly connected to the outside and can easily dissipate. But it is difficult to diffuse at higher levels. At *t* = 9000 s, the volume variation rate is close to 0 m^3^/s. There is still a hazardous gas cloud that is difficult to dissipate completely. It is mainly due to the lack of convection. This indicates that the ventilation process finally reaches a stagnant stage under the lack of convection conditions.
(5) After *t* = 4500 s in the ventilation process, differences in volume variation rates of hazardous gas clouds appear for different HBRs, which is mainly due to the layering properties of HBNG. CH_4_ and H_2_ show an uneven distribution in the vertical direction, making it easier for HBNG with a high CH_4_ proportion to diffuse outside.
(6) Extending the gas accumulation time *t*_g_ does not have a significant effect on the overall characteristics of the ventilation process. After increasing *t*_g_, the effect of different HBRs on the ventilation process remains the same as in (4). However, it affects the time point to enter the stagnation stage. As *t*_g_ increases from 3600 to 5400 and 7200 s, the ventilation time *t*_v_ into the stagnation stage increases from about 4800 s to about 5400 and 6000 s, respectively.

This work selected the evolution of the hazardous gas cloud volume to describe the diffusion and ventilation process of HBNG leakage. Compared to most previous studies, which used monitoring points and cross sections of gas distribution variations, this work can represent a more visual and comprehensive description. It can be used to support the design of ventilation and the establishment of a risk assessment system based on the hazardous gas cloud volume.

There are still some limitations in this work. Residential houses as leakage scenarios have many influencing factors and are highly random. Only studying the variation of gas cloud volume is still an insufficient reference for risk prevention and control. The actual leakage scenario has a nonuniform and nonconstant temperature field due to factors such as daylight and diurnal temperature differences. However, it is difficult to set initial and boundary conditions on such large time scales. In addition, there is a degree of uncertainty in the computational model when describing a real scenario and empirical values for the model parameters. How to quantify the impact of uncertainty on experimental and computational models is another important aspect.^29^

Combining the above points, future work will suggest considering nonconstant and complex convective conditions. For the establishment of a risk quantification system, the randomness of house need to be accommodated. At the same time, experimental and numerical calculation uncertainties were incorporated into the assessment system.

## Nomenclature

∇: Hamiltonian
*x*: displacement (m)
*p*: pressure (Pa)
*t*: solution time (s)
*U*: velocity vector (m/s)
*u*_*i*_: component of *U* (m/s)
ρ: density (kg/m^3^)
μ: dynamic viscosity (Pa·s)
*f*_*b*_: unit body force (N/m^3^)
*m*_*i*_: mass fraction
*D*_*i*_: diffusion coefficient (m^2^/s)
*R*_*i*_: component mass source
*T*: temperature (K)
λ: thermal conductivity (W/(m·K))
*c*_p_: specific heat capacity (J/(kg·K))
*k*: turbulence kinetic energy (J)
ϵ: turbulence dissipation rate
Reynolds stress
β: thermal expansion coefficient (1/K)
*v*_g_: component of velocity parallel to the gravity direction (m/s)
*u*_g_: component of velocity normal to the gravity direction (m/s)
*L*_*i*_: lower or upper explosion limited (%)
*V*_*i*_: volume fraction (%)
*U*_exit_: gas velocity at the exit (m/s)
*d*: leakage diameter (m)
ρ_∞_: ambient air density (kg/m^3^)
ρ_exit_: leakage gas density (kg/m^3^)
*t*_g_: length of time for gas accumulation (s)
*t*_v_: length of time for ventilation (s)
